# Association of social and environmental exposures at the neighborhood level with child brain volume

**DOI:** 10.1016/j.envint.2025.109576

**Published:** 2025-06-02

**Authors:** Lina V. Dimitrov, Grace M. Christensen, Benson S. Ku, Benjamin B. Risk, Anke Huels

**Affiliations:** a Department of Epidemiology, Rollins School of Public Health, Emory University, United States; b Department of Psychiatry and Behavioral Sciences, School of Medicine, Emory University, United States; c Department of Biostatistics and Bioinformatics, Rollins School of Public Health, Emory University, United States; d Gangarosa Department of Environmental Health, Rollins School of Public Health, Emory University, United States

**Keywords:** Air pollution, Area deprivation, Brain, Children, Neighborhood exposome

## Abstract

**Background:**

Prior studies show that neighborhood disadvantage negatively impacts children’s cognitive function and brain volume. However, despite their co-occurrence, there is a lack of cohesive examination of the joint effects of environmental and social factors on brain volumes.

**Objectives:**

Using environmental mixture methods, we investigated the association between a child’s neighborhood environment and brain volume in regions relevant for cognitive and cognitive-affective function.

**Methods:**

We leveraged a sample of 8574 nine and ten-year-old children from the Adolescent Brain Cognitive Development study. Exposures included 22 social and 7 environmental exposures linked to participants’ baseline addresses. Neighborhood exposome profiles were represented using self-organizing maps. Baseline cortical and subcortical volumes in 16 regions were analyzed using linear mixed effects models with neighborhood exposome profile as a categorical exposure, adjusting for individual-level demographic variables and multiple testing. We used Weighted Quantile Sum (WQS) regression to examine the total mixture effect and the relative contribution of each individual exposure to the total mixture effect.

**Results:**

Participants living in profiles defined by moderate neighborhood deprivation, high ozone, and poor walkability had greater right caudal middle frontal gyrus volumes compared to those in the reference cluster (beta: 0.11; 95% CI: 0.05, 0.17). WQS indicated that this association was primarily driven by home value and ozone concentration.

**Discussion:**

We identified multiple social and environmental factors in association with greater right caudal middle frontal gyrus volume. These results provide evidence of how multiple facets of the neighborhood environment can impact the developing brain and potentially increase the risk of future psychopathology.

## 1. Introduction

Many aspects of normal brain development in children, as well as early signatures of abnormal brain development, are still poorly understood. However, it is known that the external environment in which children grow up can be a major determinant of general future health (Braveman et al., 2011). The external environment is a major determinant of adolescent health (Viner et al., 2012). An improved understanding of the ways in which the external environment can affect a child’s developing brain can be used to inform policy initiatives that aim to improve children’s neurodevelopmental and mental health outcomes. There is emerging evidence that childhood socioeconomic status (SES) affects the pace of brain development, which in turn affects efficiency of cortical networks (Tooley et al., 2021). Furthermore, there is evidence that early life stress can accelerate the development of certain brain regions (Tottenham et al., 2010; Lupien et al., 2011). Recent studies have demonstrated the detrimental effects of neighborhood disadvantage on cognitive and cognitive-affective function in children and brain volumes of regions believed to be critical for these functions (Taylor et al., 2020; Dumornay et al., 2023). For example, neighborhood poverty has been found to account for differences in brain volumes in the prefrontal cortex and right hippocampus (Taylor et al., 2020) and neighborhood disadvantage as quantified by the Area Deprivation Index has been associated with differences in brain volume in the pars triangularis and the insula (Dumornay et al., 2023), both implicated in emotional response to stress.

Children and adolescents tend to be simultaneously exposed to multiple exposures, for example lower income communities with higher social vulnerability tend to also be the communities more likely to be located near toxic waste sites (Trottier et al., 2025). However, prior studies tend to estimate the effect of a single exposure (Ku et al., 2024), which may mischaracterize combined impacts. In particular, there is a lack of cohesive examination of both external environmental (e.g. air pollution exposure) and social (e.g. neighborhood poverty level) characteristics together (Parenteau et al., 2024; Ku et al., 2022; Ku et al., 2022), despite the importance of examining the joint effects of these co-occurring exposure types given identified interactions between social and environmental exposures on health outcomes (Ashrap et al., 2021; Eick et al., 2022; Li et al., 2022). Analyzing the neighborhood exposome, which is defined as the range of neighborhood-level environmental and social exposures that co-occur and interact to impact health, may more accurately characterize the impacts of the external environment on brain development.

The current study aims to fill the existing gaps in the literature by leveraging neighborhood factors and brain imaging data from the Adolescent Brain Cognitive Development (ABCD) study, the largest long-term study of brain development and child health in the United States. To elucidate the relationship between the neighborhood exposome and brain development., we applied novel environmental mixture techniques to 1) identify patterns of exposures in the neighborhood exposome, 2) estimate associations between different “neighborhood exposome profiles” and regional brain volumes, and 3) determine the most influential components of the neighborhood exposome on child brain volume.

## 2. Methods

### 2.1. Study population

We used tabulated data from ABCD 4.0. The ABCD study is a population-based, longitudinal study of a large and diverse sample of children in the United States, with almost 12,000 children enrolled at 9–10 years of age from 21 metropolitan areas across the United States. General inclusion and exclusion criteria for the ABCD study have been described elsewhere (Garavan et al., 2018; Karcher and Barch, 2021; Compton et al., 2019). The study includes annual follow-up to examine cognitive, behavioral, and neuroimaging outcomes over time in response to a wide range of environmental exposures. We examine baseline data collected between October 2018 and September 2019 to explore associations between the neighborhood environment and baseline measures of regional brain volume. We excluded children who had lived at their current residence for less than 1 year (n = 2129) in order to capture an appropriate exposure window (e.g. a child living at a residence for one month does not represent an etiologically relevant period of exposure to the neighborhood characteristics associated with that address), children who did not have complete baseline data on all exposures, outcomes, and covariates (n = 906), and children who did not pass T1 weighted image (MRI) data QC (n = 267), leaving us with an analytic sample of 8574 children (Supplemental Fig. S1).

### 2.2. Demographic characteristics

Demographic data for children was gathered from a survey completed by the participant’s parents. Information was collected on the child’s sex, race, and ethnicity, as well as on parents’ highest level of education and income, among other demographic variables. We categorized race into White, Black, American Indian or Alaskan Native, Asian, Pacific Islander, Some Other Race, or Two or More Races, to align with the current US Census categorization. Ethnicity was categorized as Hispanic vs. non-Hispanic. Parents’ highest level of education was collapsed for ease of interpretability into “Less Than College,” “Some College,” “Associate’s Degree,” “Bachelor’s Degree, “Master’s Degree,” or “Professional/Doctoral Degree.” The highest level of education for the child’s parents was based on the education level of the parent with the highest education level, and when education level was missing for one parent, the other parent’s education level was used. Household income was categorized as less than $50,000, $50,000–$99,999, and $100,000 or greater. We also created a “Ratio of Household Income to Poverty Level” variable, which measures the ratio of the household income per family size and the poverty level income for that household size (Rakesh et al., 2022) (e.g. a “Ratio of Household Income to Poverty Level” value of 1 means that the family was living right at the poverty level based on their household income and number of household members).

### 2.3. Exposure measurements

Several environmental and social neighborhood characteristics were captured in our neighborhood exposome profiles and assigned to the participants’ residential addresses. The participant’s caregiver was asked to provide the primary residential address during the baseline visit and at each follow-up visit. The latitude and longitude of the residential address were then geocoded to allow for data linkage with a variety of data types. ABCD data is shared with the linked data already available, and the full procedure has been previously described (Fan, 2021).

Environmental neighborhood characteristics included air pollution, proximity to roadways, lead risk, and industrial pollutants and hazardous waste sites. The following three air pollutants provided as one-year averages measured in 2016 were included: fine particulate (PM_2.5_), nitrous dioxide (NO_2_), and ozone (O_3_). Estimates for these air pollutants were calculated by ABCD using a hybrid spatiotemporal model at a 1 × 1 km^2^ spatial resolution, which combines satellite-based aerosol optical depth models, land-use regression, and chemical transport models (Di, 2020; Di et al., 2019; Requia et al., 2020). We also included an estimated measure of proximity to roadways of the residential address in 2016. Lead risk, drawn from the Washington Tracking Network, was included as an “estimate of lead exposure based on age of homes and poverty levels in census tract,” with annual averages from 2010 to 2014 (Fan, 2021). An estimate from 2015 of industrial pollutants and hazardous waste sites in close proximity to the residence was used from the Child Opportunity Index (COI) 2.0, as an indicator of potential exposure to harmful chemicals (Acevedo-Garcia et al., 2020).

Social neighborhood characteristics were derived primarily from the Area Deprivation Index (ADI), which is a commonly used measurement of neighborhood deprivation obtained from the American Community Survey 2010–2014 (Kind and Buckingham, 2018). We also included measures of access to food and green space and walkability of a neighborhood from the COI (Kind and Buckingham, 2018). To assess neighborhood ethnic and immigrant composition, we used 2014–2018 estimates from the Social Vulnerability Index (SVI) which reported on percentage minority population and percentage of persons at least 5 years of age who speak English “less than well.” A 3-year average starting from 2010 of total crime rates was derived from county level counts of arrests and offences. Further details on exposure variables are included in Supplemental Table 1.

### 2.4. Magnetic resonance imaging (MRI) of brain regions

Structural MRI data were collected by the ABCD consortium which has established an optimized acquisition process for MRI data, that is harmonized to be compatible across Siemens, General Electric, and Philips scanners, as previously described. Imaging methods and assessment are described in greater detail elsewhere (Casey et al., 2018; Hagler et al., 2019). In selecting our outcome measures, we decided *a priori* to examine the following 16 lateralized brain regions relevant for cognitive and cognitive-affective function based on previous work in the ABCD cohort examining neighborhood adversity and brain volume in children (Taylor et al., 2020; Dumornay et al., 2023): the amygdala, hippocampus, superior frontal gyrus, caudal anterior cingulate cortex, caudal middle frontal gyrus, lateral orbitofrontal cortex, medial orbitofrontal cortex, pars opercularis, pars triangularis, pars orbitalis, rostral anterior cingulate cortex, rostral middle frontal gyrus, frontal pole, and insula. Gray matter volume of cortical regions of interest were based on the Desikan-Killiany atlas (Desikan et al., 2006) and gray matter volume of subcortical regions were based on the aseg atlas (Hagler et al., 2019). All brain region volumes were converted to z-scores for easier interpretation and comparability across brain regions.

### 2.5. Statistical analysis

**Fig. 1.**
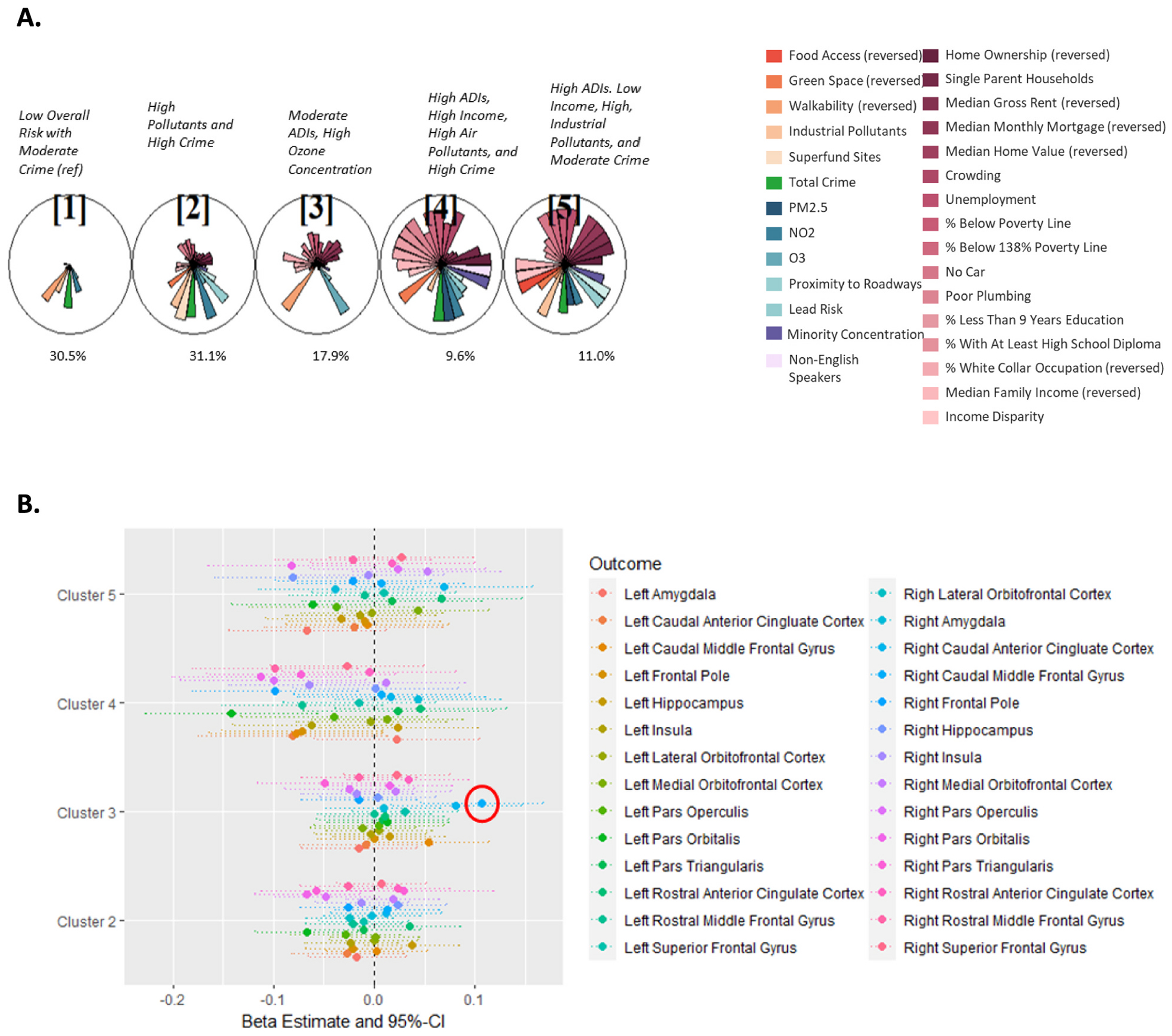
Associations between neighborhood exposure profiles and brain volume. A. SOM cluster star plot, where slices represent median values of a mixture component. Each circle represents a SOM cluster/neighborhood-level exposure profile that a child is exposed to. Pink gradient slices correspond to Area Deprivation Index (ADI) variables, orange gradient slices correspond to Child Opportunity Index (COI) variables, green slices correspond to total crime, blue gradient slices correspond to environmental exposures, and purple gradient slices correspond to minority and non-English speaker concentration. All variables were coded such that “more means worse.” Cluster 1 is the reference cluster. B. Forest plot depicting association estimates between SOM clusters and brain volume and 95 % CI in 16 lateralized brain regions. Mixed effects linear models were used for each outcome, with adjustment for family relatedness as a random effect and intracranial volume, age, sex, race, ethnicity, and highest level of parent education as fixed effects. Right caudal middle frontal gyrus (circled in red) volume was significantly associated with Cluster 3 after correcting for multiple testing using the Bonferroni correction. (For interpretation of the references to colour in this figure legend, the reader is referred to the web version of this article.)

We used self-organizing maps (SOM) to derive exposure profiles indicative of a child’s neighborhood exposure profile based on their recorded residence. SOM is an unsupervised machine learning approach that creates class profiles (i.e. clusters) by maximizing intra-cluster homogeneity and minimizing inter-cluster homogeneity. We used the SOM algorithm (Pearce et al., 2014; Pearce et al., 2016) as implemented in https://github.com/johnlpearce/ECM to identify clusters with similar environmental and social characteristics based on the exposure measurements described above. The SOM algorithm led to the selection of the 5 exposure profiles presented in Fig. 1A. The selection of these clusters was based on identification of group structures using within cluster sum of squares and between cluster sum of squares statistics, assessment of sample distribution across clusters, and visual evaluation of cluster star plots. Participants were then assigned an SOM cluster based on their relevant exposure characteristics and the SOM clusters were used as a categorical exposure in linear mixed-effects regression models, with one model per outcome. A directed acyclic graph (DAG, Fig. S2) was used to select confounders (Fig. S2). Our models included race, ethnicity, and highest level of parent education as potential confounders of the association between environmental and social neighborhood exposures and brain volume, and age, sex, and intracranial volume were included in all models as predictors of the outcome. The latter variables were modeled as fixed effects, while family relatedness was included in the models as a random effect. Variance inflation factors were examined for the exposure and covariates to avoid multi-collinearity issues (Supplemental Table S8). Due to the high amount of missingness in the family income variable, we did not adjust for this in our main analyses but conducted sensitivity analyses in which we further adjusted for this variable. We also conducted sensitivity analyses to assess whether associations were similar when excluding children receiving any psychiatric medications. (Parents were asked what medications their children had taken in the past 2 weeks. Participants taking medications commonly prescribed to treat mental illnesses were excluded in sensitivity analysis. A full list of medications used for exclusion is available in the supplementary materials.) We additionally conducted sensitivity analyses of our main finding among subgroups of children living at their current residence for 2+, 4+, and 8+ years. Determination of statistically significant associations between SOM clusters and brain regions was based on a Bonferroni-adjusted p-value of 0.003125 (adjusted based on an alpha level 0.05 and the number of brain regions being assessed (16)).

For brain region volumes significantly associated with a particular SOM profile, we used Weighted Quantile Sum regression (WQS) to investigate the contribution of individual components to the associations of brain regions with certain exposure profiles. WQS allows for both an estimate of the total mixture effect of increasing all exposures by one quantile on the outcome, as well as an estimate of the relative contribution of exposures within the mixture while appropriately handling highly correlated exposures (Carrico et al., 2015). We used the *gWQS* (version 3.0.5) package in R for these analyses. Our WQS model was adjusted for the same fixed effect covariates as the mixed effects linear regression models; WQS does not allow for inclusion of a random effect term. However, since our primary aim in utilizing WQS was to understand the contribution of mixture components to the observed overall mixture effect, this limitation of WQS was not a concern since WQS does not calculate a standard error for the weights.

## 3. Results

### 3.1. Population characteristics

The analytic sample of 8574 children was approximately evenly split between males and females. The median age was 9.92 years, and the majority of children were White (67 %), with Black children as the next most prevalent racial group in the sample (14 %). 62 % of families in the sample had at least one parent with a bachelor’s degree or higher, and 13 % and 12 %, respectively, with an Associate’s degree or some college. Family income distribution was approximately split into thirds between those making less than $50,000, those making $50,000–$99,999, and those making $100,000+ annually (Table 1). Median and IQR for baseline measures of brain volume in each of the examined regions are shown in Table 2. All brain region volumes were weakly to moderately positively correlated with each other and with intracranial volume (Fig. S3).

Neighborhood exposures were correlated with one another, underscoring the importance of considering their joint effects. PM_2.5_ (median: 7.66 μg/m^3^, IQR: 6.54, 8.60) and NO_2_ (median: 18.8 ppb, IQR:14.7, 22.2) were positively correlated (r = 0.21), while O_3_ (median: 40.6 ppb, IQR:38.4, 45.4) and PM_2.5_ were negatively correlated (r = −0.16). Lead risk (median: 17, IQR:7, 32) was also positively correlated with PM_2.5_ (r = 0.18) and NO_2_ (r = 0.23), but not O_3_ (r = −0.002). Walkability (median: 10.8, IQR: 7.3, 14.2) was positively correlated with all air pollutants, although more strongly correlated with PM_2.5_ (r = 0.27) and NO_2_ (r = 0.42) than with O_3_ (r = 0.05). PM_2.5_ was negatively correlated with favorable nSES variables (e.g., percent home ownership) and positively correlated with adverse nSES variables (e.g., percent living below the poverty level). NO_2_ followed the same pattern as PM_2.5_ but was less strongly correlated with nSES variables overall. O_3_ was not strongly correlated with any nSES variables but was weakly positively correlated with median home value (r = 0.06), median gross rent (0.11), and median monthly mortgage (r = 0.09). All nSES variables were correlated with each other in the expected directions (i.e., positive correlations were observed between favorable nSES variables as well as between adverse nSES variables; Fig. S4).

### 3.2. Neighborhood exposure profiles

**Fig. 2.**
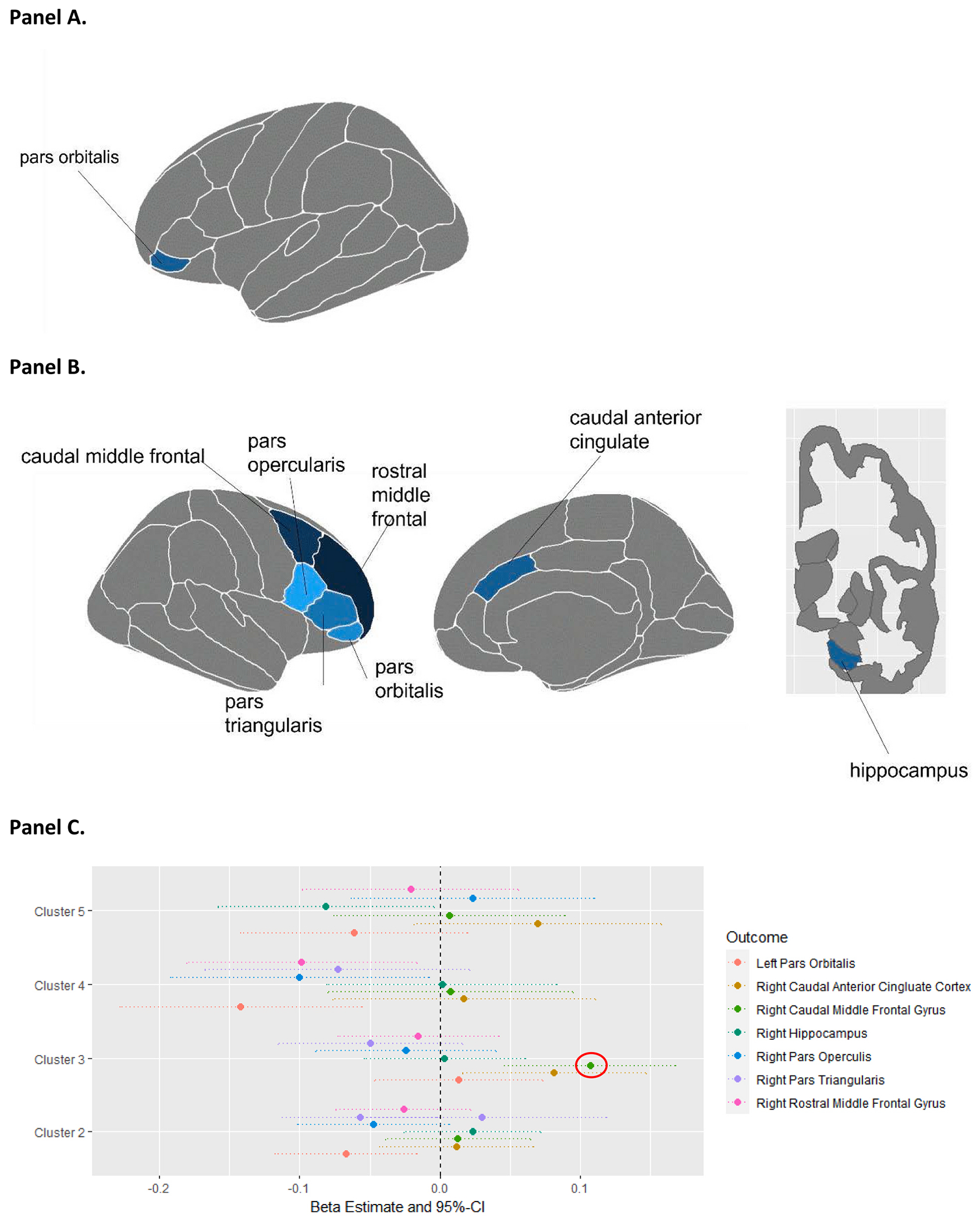
Spatial associations and effect estimates for all brain regions significantly associated with one or more cluster at an alpha level of 0.05. A. Left hemisphere regions. B. Right hemisphere regions. Left panel shows lateral view and middle panel shows medial view. Right panel shows cross-section where hippocampus is visible. C. Forest plot depicting association estimates between SOM clusters and brain volume and 95 % CI in the 7 brain regions significantly associated with one or more cluster at an alpha level of 0.05. Mixed effects linear models were used for each outcome, with adjustment for family relatedness as a random effect and intracranial volume, age, sex, race, ethnicity, and highest level of parent education as fixed effects.

The SOM identified 5 clusters of neighborhood exposure profiles based on our included environmental and social neighborhood factors (Fig. 2A). These clusters reflected the following exposure profiles: low overall risk with moderate crime (Cluster 1); high pollutants and high crime (Cluster 2); moderate neighborhood deprivation (represented by ADI variables) with high ozone concentration, and poor walkability (Cluster 3); high neighborhood deprivation, moderate wealth, high industrial pollutants, and high crime (Cluster 4); and high neighborhood deprivation, low income, high industrial pollutants, and moderate crime (Cluster 5). Cluster 1 was used as the reference cluster in regression modeling as this cluster had the lowest adverse exposures compared to other clusters.

Participants were unevenly distributed across clusters, with about a 3rd each in Clusters 1 and 2 and 18 %, 10 %, and 11 % in Clusters 3 through 5, respectively (Table 1). The distribution of sex and the median age across clusters was relatively equal. The majority of children in Clusters 1 through 3 were White, with Cluster 1 having the largest percentage of White children at almost 80 %. Cluster 4 was about evenly split between White residents and residents of other racial groups, while Cluster 5 had a majority non-White population, with 56 % of Cluster 5 identifying as Black. Cluster 4 had the highest percentage of families of Hispanic ethnicity at 70 %, followed by Cluster 3 with about a quarter of Hispanic ethnicity. Cluster 1 was the most highly educated, with about a third of parents obtaining bachelor’s degrees and almost 20 % obtaining professional/doctoral degrees. Clusters 4 and 5 had the lowest education rates, with more than a third of parents having only a high school education or less. Cluster 1 was also the wealthiest, with almost 50 % of families making $100,000 or more annually. Only around a 3rd of the sample in Clusters 2 and 3 fell into this income bracket, and the majority of families in Clusters 3 and 4 made less than $50,000 annually. These patterns are also reflected in the household income-to-poverty ratio for each cluster, with the median score in Cluster 5 being 1.2 (indicating living almost exactly at the poverty line), while the median household income-to-poverty ratio in Cluster 1 was 5.2 (Table 1). Median and IQR baseline measures of brain volume are presented by cluster in Table 2.

### 3.3. Associations between neighborhood exposure profiles and brain volume

When SOM clusters were modeled as a categorical exposure in linear mixed models, after accounting for individual demographic characteristics and total intracranial volume, multiple brain regions trended toward lower volumes in Cluster 4, the cluster characterized by high neighborhood deprivation, moderate wealth, high industrial pollutants, and high crime, as compared to Cluster 1. Among these, the left pars orbitalis had the strongest negative relationship with Cluster 4 (beta: −0.14, interpreted as, on average, a 0.14 of a standard deviation lower volume in Cluster 4 compared to Cluster 1, 95 % CI: −0.23, −0.06; Fig. 1B, Table S3, Fig. S5B).

In addition to the left pars orbitalis, six other brain regions were significantly associated with one or more SOM clusters at a nominal alpha level of 0.05. The right pars triangularis and left pars orbitalis were negatively associated with Cluster 2, the cluster characterized by high pollutants and high crime. Both the right caudal middle frontal gyrus and the right caudal anterior cingulate cortex were positively associated with Cluster 3, the cluster characterized by moderate neighborhood deprivation (represented by ADI variables) with high ozone concentration, poor walkability, and low crime. The right hippocampus, right pars opercularis, left and right pars orbitalis, and right rostral middle frontal gyrus were all negatively associated with Cluster 5, the cluster characterized by high neighborhood deprivation, low income, high industrial pollutants, and moderate crime (Table S3, Fig. S5).

After correcting for multiple testing using the Bonferroni correction, the only significant association between cluster and brain region volume was the association between Cluster 3 and right caudal middle frontal gyrus volume; Cluster 3 was associated with a larger right caudal middle frontal gyrus volume compared to Cluster 1 (beta: 0.11; 95 % CI: 0.05, 0.17; Fig. 2B, Table S4). These findings were consistent both after additional adjustment for household income to poverty ratio and after restriction to children not taking any psychiatric medications (Tables S5 and S6). Given the fact that our sample is restricted to those who have lived at their current residence for at least one year and includes a heterogeneous mix of children with regards to time living at their current residence, we also performed a sensitivity analysis of our main finding with restrictions on duration living at current address. While subgroup sample sizes were too small to restrict to an exact duration (e.g. *exactly* 4 years at current residence), we reran the right caudal middle frontal gyrus adjusted model among children living in their current residence for 2+, 4+, and 8+ years. All results were consistent with our main finding, the effect was even stronger among children living at their current residence for 8+ years, although not statistically significant given the large reduction in sample size (Table S7).

**Fig. 3.**
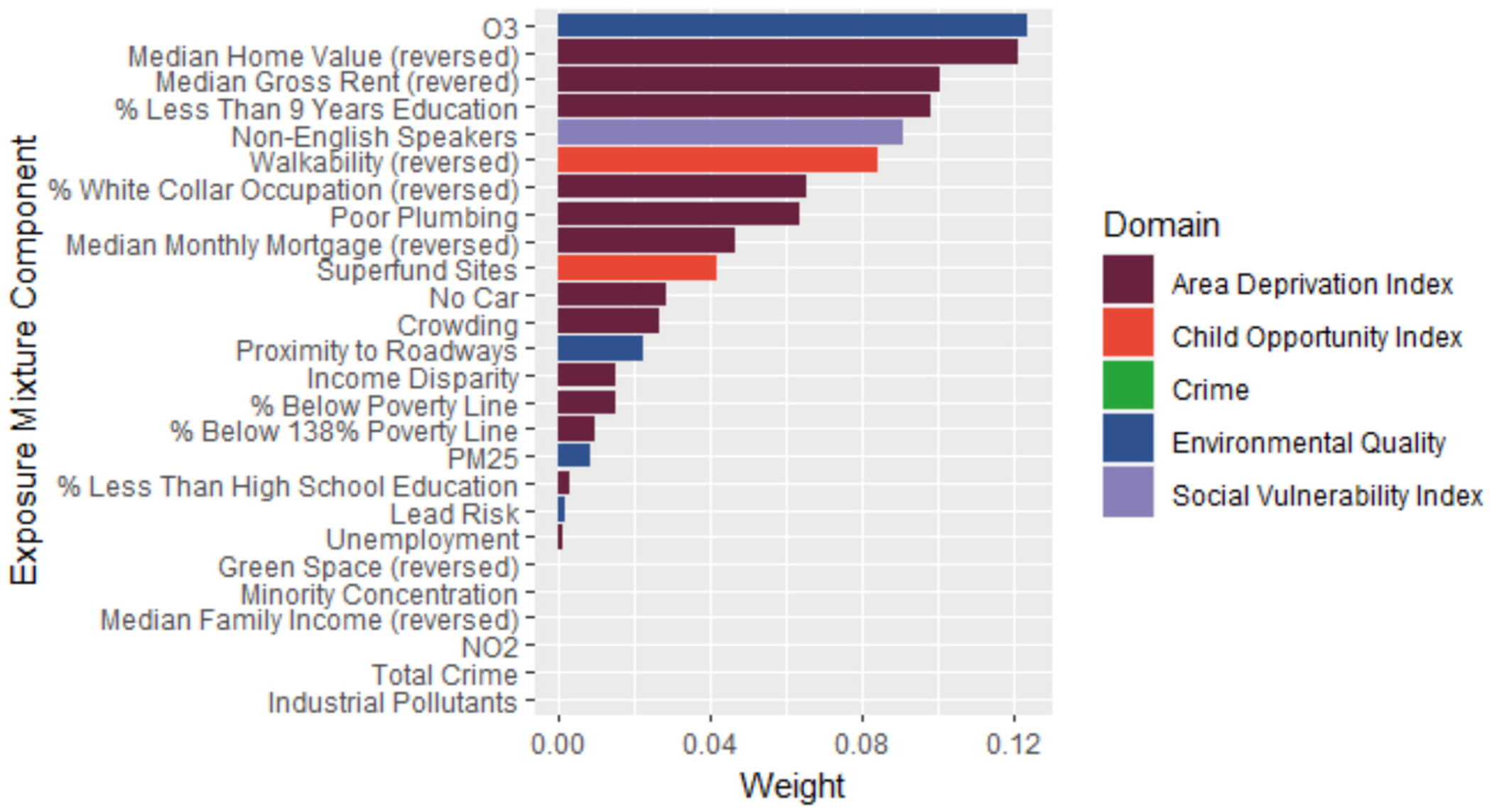
Weighted quantile sum regression mixture component weights for the association between neighborhood-level exposures and right caudal middle frontal gyrus volume. Individual-level covariates included in the model were intracranial volume, age, sex, race, ethnicity, and highest level of parent education. Bar lengths/weights represent the relative contribution of each mixture component to the overall positive mixture effect. Overall positive mixture effect: 0.04; 95 % CI: 0.01, 0.06.

Next, we used a positive constraint weighted quantile sum regression to better understand which exposures within the Cluster 3 exposure profile might be driving the significant association observed between this cluster and right caudal middle frontal gyrus volume. Overall, the effect of increasing all exposures simultaneously by one decile corresponded to close to a 0.04 standard deviation increase in brain volume in the right caudal middle frontal gyrus (beta = 0.04, 95 % CI: 0.01, 0.06). The largest weights, in order, corresponding to the contribution of each exposure to the total positive effect were ozone, median home value (reversed), and median gross rent (reversed; Fig. 3). These weights align with what we observed in the SOM clusters; Cluster 3 had the second lowest median home value and median gross rent after Cluster 5, and Cluster 3 had the highest ozone concentration of all the clusters.

## 4. Discussion

In this study, we investigated the association between regional brain volumes relevant for cognition and emotion regulation and children’s neighborhood environments, leveraging data from 8574 children enrolled in the ABCD study. Using a combination of novel environmental mixture techniques, we identified a statistically significant association between living in neighborhoods characterized by moderate neighborhood deprivation, high ozone concentration, and poor walkability and larger right caudal middle frontal gyrus volume. The combination of heightened ozone levels and lower home value contributed the most to the observed association. Utilizing multiple mixture methods allowed us to gain insight into specific exposure patterns that may be relevant for right caudal middle frontal gyrus maturation, reflecting the neighborhood exposome in a more comprehensive way than possible with single exposure analyses.

Although no studies to date that we are aware of have specifically reported on associations between ozone exposure in children and the right caudal middle frontal gyrus, our findings do align with prior literature suggesting the adverse effects of ozone on neurodevelopment generally. A study conducted in this same cohort found that higher ozone concentrations were associated with greater intra-network, but less subcortical-to-network functional connectivity over time (Cotter et al., 2023). Another recent population-based case-control study focused on autism spectrum disorder has linked prenatal ozone exposure to poor neurodevelopmental outcomes (Goodrich et al., 2024). Therefore, our findings add to a nascent body of evidence that suggests that ozone exposure affects brain development in children.

In terms of the finding that home value is another top driver of the noted mixture effect on right caudal middle frontal gyrus volume, home value, in this case, is likely functioning as a marker of a generally lower resourced area. This interpretation aligns with prior literature that suggests an influential role of neighborhood socioeconomic disadvantage on brain structure and neurodevelopment (Hackman et al., 2021). For example, a study using both objective census-level measures of neighborhood deprivation and neighbor self-reported perceptions of neighborhood deprivation found that neighborhood disadvantage was associated with greater right amygdala reactivity to threat when there was a perception of greater permissiveness in regards to safety (Suarez et al., 2022). Additionally, neighborhood disadvantage has been linked to lower cortical thickness in multiple brain regions in children (Rakesh et al., 2022).

Current evidence suggests that early life adversity does not have a simple, uniform impact on brain volume, but rather functions in age-, experience-, and brain region-specific ways to impact the developing brain (Vannucci et al., 2023). Although, historically, larger brain volume has often been connected to positive health outcomes, the stress acceleration hypothesis (Callaghan and Tottenham, 2016) posits that chronic stress may result in faster maturation of the brain in some regions, which may explain the current observation of larger right caudal middle frontal gyrus volumes in children from more adverse exposome profiles than the reference profile. Meta-analytic evidence demonstrates an association with interpersonal adversity and initially larger brain volumes in frontolimbic regions until about 10 years of age, after which these exposures become associated with increasingly smaller volumes (Vannucci et al., 2023). Interestingly, the right caudal middle frontal gyrus, the singular brain region of interest that withstood statistical significance correction for multiple testing in our study, is part of the frontolimbic system. Furthermore, our sample of children falls into an age range aligning with this latter *meta*-analytic finding, as the median age of our sample is 9.9 years, and the age range of our sample is 8–11 years. Although our study examined area-level adversity rather than interpersonal adversity, it is possible that area-level adversity independently affects this brain region in a mechanistically similar way to interpersonal adversity. It is also possible that the kind of external environment represented by high pollution and neighborhood deprivation may be associated with greater familial stress and adversity, whether casually or by correlation. Prior studies have also demonstrated curvilinear age-related changes in children from low socio-economic status (SES) backgrounds, while trajectories of children from higher SES backgrounds showed a linear pattern; (Piccolo Lda et al., 2016) children from low SES backgrounds also showed earlier peaks in brain development, later followed by earlier cortical thinning (Piccolo Lda et al., 2016). More recent findings have also extended these observations into the realm of functional implications, such as a study showing that greater cortical thickness in the right caudal middle frontal region mediates the association between greater pre-school age cortisol reactivity and lower school-age executive function (Feola et al., 2020). Thus, it is possible that high levels of stress, and in turn increased cortisol reactivity, may affect the developmental trajectory of the prefrontal cortex, leading to earlier peaks in brain maturation which then result in downstream functional impacts (Feola et al., 2020).

The right caudal middle frontal gyrus is also a brain region that corresponds to the right posterior dorsolateral prefrontal cortex, a brain region which has been implicated in schizophrenia etiology (Volpe et al., 2012). Of note, there is a known link between urban upbringing and schizophrenia (Paksarian et al., 2018; Vassos et al., 2012). In addition, there have been studies demonstrating the associations between an urban upbringing and anatomical alterations in the dorsolateral prefrontal cortex (Ku et al., 2022; Haddad et al., 2014). Our prior findings in this same cohort demonstrate an association between the same exposome profile that was identified here in association with larger right caudal middle frontal gyrus volumes (characterized by moderate neighborhood deprivation, high ozone concentration, and poor walkability) and persistent distressing psychotic-like experiences (PLE) in children (Ku et al., 2025). Although some level of PLEs appears to occur in the general population, PLEs that are persistent and distressing in nature have been shown to impact psychological burden in adolescence and lead to impairments in functioning (Mylona et al., 2022; Karcher et al., 2022). The alignment of our findings with this prior finding relating similar area-level contextual factors to persistent distressing PLEs suggests the importance of examining the neighborhood exposome in relation to psychosis and psychotic disorders, and warrants further investigation.

Beyond our statistically significant findings, we also observed notable trends related to other brain regions and neighborhood exposome profiles. While it is important to not over-interpret these findings at this stage, we highlight them here as potential avenues for future directions of investigation. Although these associations did not survive multiple testing correction, the neighborhood exposome profile characterized by high neighborhood deprivation, moderate wealth, high industrial pollutants, and high crime (Cluster 4) was associated with smaller volumes in three brain regions at an uncorrected p-value threshold of 0.05—the right rostral middle frontal gyrus, right pars operculis, and left pars orbitalis. These findings also align with a general trend toward smaller brain volumes across regions in Cluster 4 compared to the reference cluster. Although there is a paucity of literature focusing on these brain regions, the general tendency of smaller regional brain volumes to often represent functional deficits may suggest that adversity related to high neighborhood deprivation, even in areas with higher property value, could be detrimental to these brain regions, but more research is needed to evaluate this further, particularly as different types of adversity have been known to affect brain volume differentially (LoPilato et al., 2019). When using an uncorrected p-value threshold of 0.05, we also observed an association between the neighborhood exposome characterized by high neighborhood deprivation, low income, high industrial pollutants, and moderate crime (Cluster 5) and smaller right hippocampal volume, but not left hippocampal volume. Meta-analytic evidence from studies examining childhood maltreatment, specifically in individuals with post-traumatic stress disorder (PTSD), suggests that hippocampal volume deficits associated with childhood maltreatment appear in adulthood (Woon and Hedges, 2008). In addition, a small study of 49 children found that children who experienced early-life adversity (defined as maltreatment and out-of-home placement) had smaller hippocampal volumes during childhood than their unexposed counterparts (Dahmen et al., 2017). Larger studies have demonstrated the inverse relationships between cortisol, but not perceived lifetime stress, and hippocampal volume among youth at clinical high risk for psychosis (Aberizk et al., 2024). While a previous study in the ABCD cohort did not find an association between neighborhood deprivation and overall hippocampal volume when examining the hippocampus as a whole rather than laterally (Dumornay et al., 2023), another study conducted in this same cohort found that greater neighborhood poverty was associated with reductions in right hippocampal volume (Taylor et al., 2020), consistent with our findings. These disparate results may be due to masking of effects when examining the hippocampus in a non-lateralized fashion, as was done in the former study. Furthermore, normalization of brain volumes by dividing by intracranial volume rather than adjusting for this in the model, as was done in the latter study, may have also contributed to different findings. Generally, our findings align with evidence from animal models and human studies that suggest that early-life environmental stressors appear to have a greater impact on brain morphometry in the right hemisphere (Esteves et al., 2021).

Our study has several strengths. First, our large sample size allowed us to make estimates with relatively good precision. Second, our approach to considering the holistic neighborhood exposome, rather than focusing on one exposure or one type of exposure at a time, allowed us to gain a more nuanced understanding of relevant exposures among many highly correlated exposures. Furthermore, triangulation of two environmental mixture methods allowed us to discern which exposures are elevated in certain clusters versus when this elevation is actually pertinent for the outcome of interest. For example, when visually inspecting the SOM star plot for Cluster 3, this cluster is notably defined by spikes in both ozone concentration and poor walkability. However, the weight plots from WQS regression elucidate that, while ozone *is* a key driver of the observed association, several of the mid-level ADI variables play a greater role in the observed overall mixture effect than does walkability. Lastly, our use of the DAG framework to select relevant individual-level confounders allowed us to appropriately isolate the effect of the neighborhood exposome on brain volume independent of individual characteristics and without potential inappropriate adjustment for mediators or colliders that would bias the results.

Our study is subject to some limitations. Since our study included only brain volume measurements at a single timepoint (baseline visit) to determine associations between the neighborhood exposome and regional brain volumes, we could not evaluate the impact of the neighborhood exposome on developmental trajectories over time. We also did not have adequate sample size to break children down into subgroups based on duration of living at current residence for our main analysis. Furthermore, even though most of our exposure measurements occurred prior to the brain volume measurements, it is not always clear what the relevant exposure window may be. Additionally, we were not able to control for average annual temperatures and other meteorological factors to better isolate the effects of pollutants such as ozone. Also, while our focus here was on brain morphology, future studies should link neighborhood factors, regional structural brain morphology, and cognitive and behavioral measures. Lastly, it is possible that this research is subject to prevalent exposure bias. Prevalent exposure bias can occur when participants in the analytic sample are already exposed at the time of recruitment, as opposed to inclusion of only participants with incident exposure in the analyses. If the exposure is harmful, this may cause a bias of the effect estimate towards the null, as it is possible participants who experienced severe outcomes that may prevent them from study participation would not be captured in the analytic sample, leaving an analytic sample of relatively healthier individuals. We intentionally included participants with prevalent exposure in our analytic sample (i.e. by stipulating in the inclusion criteria that participants must have resided at their current address for at least one year) in order to provide some assurance that neighborhood exposome profiles assigned to each participant actually reflect true exposure patterns. For example, if someone had only moved to their baseline address 2 months prior, this would not reflect the true neighborhood exposome for that individual within an etiologically relevant time frame. However, we do not have a strong reason to believe that it would be very common for the impact of the neighborhood-level exposures examined here to have such a drastic early life impact so as to preclude study participation and thus believe the impact of prevalent exposure bias on our results, if any, is minimal.

## 5. Conclusions

This study provides evidence for an association between the neighborhood exposome and right caudal middle frontal gyrus volume in children, independent of individual-level demographic and socioeconomic factors. Specifically, the neighborhood exposome profile characterized by moderate neighborhood deprivation, high ozone concentration, and poor walkability was associated with greater right caudal middle frontal gyrus volume, and ozone concentration and home value were top drivers of this association. These results provide evidence of how multiple facets of the neighborhood environment can impact the developing brain.

## Supplementary Material

1

2

Supplementary data to this article can be found online at https://doi.org/10.1016/j.envint.2025.109576.

## Figures and Tables

**Table 1:** Description of Adolescent Brain Cognitive Development (ABCD) study sample (N = 8574). A. Study sample characteristics by Self-Organizing Map (SOM) cluster. B. Environmental exposures by SOM cluster. C. Social exposures by SOM cluster.

	Total	SOM Cluster^a^				
		1	2	3	4	5
**A. Sample Characteristics**						
N (%)	8574 (100)	2642 (30.81)	2686 (31.33)	1561 (18.21)	760 (8.86)	925 (10.79)
Female Sex, n (%)	4108 (48 %)	1213 (46 %)	1288 (48 %)	775 (50 %)	373 (49 %)	459 (50 %)
Age, median (IQR)	9.92 (9.33, 10.50)	9.92 (9.42, 10.50)	10.00 (9.33, 10.50)	9.83 (9.25, 10.42)	9.92 (9.33, 10.42)	9.83 (9.33, 10.42)
Race, n (%)						
White	5710 (67 %)	2094 (79 %)	1827 (68 %)	1185 (76 %)	376 (49 %)	228 (25 %)
Black	1173 (14 %)	86 (3.3 %)	310 (12 %)	179 (11 %)	79 (10 %)	519 (56 %)
American Indian or Alaskan Native	40 (0.5 %)	8 (0.3 %)	15 (0.6 %)	4 (0.3 %)	7 (0.9 %)	6 (0.6 %)
Asian	196 (2.3 %)	90 (3.4 %)	68 (2.5 %)	11 (0.7 %)	24 (3.2 %)	3 (0.3 %)
Pacific Islander	10 (0.1 %)	4 (0.2 %)	3 (0.1 %)	0 (0 %)	3 (0.4 %)	0 (0 %)
Some Other Race	384 (4.5 %)	40 (1.5 %)	75 (2.8 %)	42 (2.7 %)	198 (26 %)	29 (3.1 %)
Two or More Races	1061 (12 %)	320 (12 %)	388 (14 %)	140 (9.0 %)	73 (9.6 %)	140 (15 %)
Hispanic Ethnicity, n (%)	1668 (19 %)	251 (9.5 %)	399 (15 %)	372 (24 %)	532 (70 %)	114 (12 %)
Highest Level of Parent Education, n (%)						
Less than college	1046 (12 %)	64 (2.4 %)	178 (6.6 %)	214 (14 %)	285 (38 %)	305 (33 %)
Some College	1024 (12 %)	173 (6.5 %)	289 (11 %)	200 (13 %)	140 (18 %)	222 (24 %)
Associate’s Degree	1102 (13 %)	206 (7.8 %)	331 (12 %)	252 (16 %)	127 (17 %)	186 (20 %)
Bachelor’s Degree	2269 (26 %)	840 (32 %)	810 (30 %)	391 (25 %)	112 (15 %)	116 (13 %)
Master’s Degree	2176 (25 %)	895 (34 %)	749 (28 %)	364 (23 %)	84 (11 %)	84 (9.1 %)
Professional/Doctoral Degree	957 (11 %)	464 (18 %)	329 (12 %)	140 (9.0 %)	12 (1.6 %)	12 (1.3 %)
Household Income, n (%)						
Less than $50,000	3015 (38 %)	774 (31 %)	782 (31 %)	511 (35 %)	397 (63 %)	551 (70 %)
$50,000–$99,999	2299 (29 %)	567 (23 %)	856 (34 %)	527 (36 %)	165 (26 %)	184 (23 %)
$100,000+	2590 (33 %)	1153 (46 %)	896 (35 %)	416 (29 %)	72 (11 %)	53 (6.7 %)
Missing income	670 (8 %)	148 (6 %)	152 (6 %)	107 (7 %)	126 (17 %)	137 (15 %)
Ratio of Household Income to Poverty Level (per family size), median (IQR)	3.6 (1.8, 6.1)	5.2 (3.6, 7.3)	3.6 (2.2, 6.1)	3.0 (1.7, 5.2)	1.5 (0.7, 3.0)	1.2 (0.5, 2.2)
**B. Environmental Exposures** Median(IQR)						
PM_2.5_ (μg/m^3^)	7.66 (6.54, 8.60)	7.15 (6.13, 8.11)	7.73 (6.66, 8.59)	7.37 (6.36, 8.13)	8.98 (8.39, 10.10)	8.49 (7.36, 9.35)
NO_2_ (ppb)	18.8 (14.7, 22.2)	17.2 (13.5, 21.6)	21.0 (18.7, 25.2)	13.1 (10.1, 15.7)	21.1 (19.1, 23.4)	19.7 (16.4, 21.9)
O_3_ (ppb)	40.6 (38.4, 45.4)	40.3 (38.0, 44.4)	40.4 (38.2, 44.8)	43.8 (39.0, 47.4)	42.8 (39.7, 45.8)	39.7 (38.4, 42.1)
Industrial Pollutants^b^	−0.40 (−0.72, 0.49)	−0.29 (−0.62, 0.44)	−0.58 (−0.85, −0.22)	1.05 (−0.15, 1.56)	0.05 (−0.54, 0.64)	−0.70 (−1.04, −0.56)
Hazardous Waste Sites^c^	0.27 (0.27, 0.27)	0.27 (0.27, 0.27)	0.27 (0.27, 0.27)	0.27 (0.27, 0.27)	0.27 (0.27, 0.27)	0.27 (0.27, 0.27)
% Lead Risk^d^	17 (7, 32)	9 (4, 19)	25 (11, 39)	9 (6, 19)	26 (15, 37)	39 (27, 50)
Proximity to Roadways^e^	841 (396, 1553)	1137 (571, 2067)	654 (327, 1147)	1052 (469, 2173)	669 (333, 1260)	601 (266, 1122)
**C. Social Exposures** Median(IQR)						
**Area Deprivation Index**						
% Single Parent Households	14 (9, 23)	8 (5, 11)	15 (11, 20)	16 (12, 22)	28 (21, 34)	38 (29, 46)
% Home Ownership,	71 (53, 84)	85 (78, 91)	65 (52, 76)	74 (62, 83)	35 (21, 51)	45 (34, 55)
% Less Than 9 Years Education	2.4 (0.9, 5.5)	1.0 (0.4, 1.9)	2.1 (1.0, 4.1)	3.7 (2.0, 6.2)	19.3 (13.8, 27.8)	5.9 (3.2, 8.9)
% With at Least High School Diploma	93 (85, 96)	97 (95, 98)	93 (90, 96)	89 (84, 93)	66 (54, 75)	81 (74, 86)
% White Collar Occupation	94.6 (91.6, 96.7)	95.1 (92.8, 97.0)	95.4 (93.3, 97.2)	91.9 (89.1, 94.9)	90.9 (87.9, 93.6)	95.4 (92.8, 97.7)
Median Family Income	73,894 (51,955, 98,229)	104,936 (92,375, 124,479)	74,171 (63,201, 87,023)	64,340 (51,197, 73,667)	40,152 (31,541, 48,503)	35,655 (26,777, 42,857)
Income Disparity^f^	2.00 (1.25, 2.86)	0.92 (0.33, 1.47)	2.08 (1.63, 2.61)	2.32 (1.80, 2.91)	3.14 (2.55, 3.66)	4.01 (3.45, 4.69)
Median Home Value	227,000 (150,050, 318,800)	305,800 (244,550, 421,000)	220,600 (171,225, 313,975)	160,700 (120,800, 215,800)	261,850 (186,100, 318,375)	77,700 (58,300, 107,000)
Median Gross Rent	1054 (856, 1326)	1373 (1110, 1680)	1010 (869, 1222)	923 (770, 1140)	1044 (932, 1160)	751 (639, 844)
Median Monthly Mortgage	1403 (1070, 1731)	1712 (1476, 2101)	1366 (1141, 1654)	1130 (899, 1367)	1393 (1,134, 1751)	769 (617, 948)
% occupied housing units with >1 person per room (crowding)	1.6 (0.5, 3.8)	0.7 (0.0, 1.7)	1.5 (0.5, 3.3)	2.1 (0.8, 3.8)	16.9 (10.0, 25.5)	2.4 (0.9, 4.7)
% Unemployment	7.4 (5.0, 11.0)	5.2 (3.7, 7.0)	7.1 (5.2, 9.5)	8.5 (5.8, 11.3)	12.4 (9.9, 16.0)	15.5 (11.5, 21.5)
% Below Poverty Line	7 (3, 15)	2 (1, 4)	7 (4, 11)	10 (6, 15)	23 (16, 33)	26 (19, 37)
% Below 138 % Poverty Line	16 (9, 29)	7 (4, 9)	16 (12, 22)	20 (15, 28)	41 (32, 51)	43 (34, 53)
% Households with No Car	5 (2, 10)	2 (1, 4)	6 (4, 10)	4 (3, 7)	12 (7, 19)	23 (15, 36)
% Poor Plumbing	0.00 (0.00, 0.32)	0.00 (0.00, 0.00)	0.00 (0.00, 0.00)	0.00 (0.00, 0.44)	0.00 (0.00, 0.87)	0.00 (0.00, 0.64)
**Child Opportunity Index**						
Access to Food^g^	0.37 (−0.19, 0.67)	0.56 (0.34, 0.73)	0.32 (−0.17, 0.63)	0.19 (−0.20, 0.49)	0.58 (−0.09, 0.85)	−1.85 (−3.26, −0.51)
Access to Green Space^h^	0.33 (−0.93, 0.56)	0.38 (−0.25, 0.87)	−0.71 (−1.10, −0.33)	0.77 (0.03, 1.08)	−1.61 (−1.99, −1.18)	−0.80 (−1.30, −0.38)
Walkability^i^	10.8 (7.3, 14.2)	7.8 (6.2, 11.0)	13.5 (10.5, 15.3)	7.3 (5.3, 9.7)	14.2 (12.7, 15.5)	12.3 (9.3, 15.0)
Total Crime	22,761 (7832, 53,400)	18,253 (8163, 35,489)	32,424 (19,950, 77,667)	517 (0, 3914)	107,316 (53,400, 348,049)	35,489 (22,761, 45,073)
**Social Vulnerability Index**						
Percentile of % Minority Population	0.48 (0.29, 0.74)	0.33 (0.19, 0.49)	0.47 (0.32, 0.63)	0.45 (0.22, 0.77)	0.92 (0.82, 0.96)	0.80 (0.69, 0.91)
Percentile of % Non-English Speakers	0.50 (0.28, 0.74)	0.39 (0.24, 0.57)	0.54 (0.34, 0.70)	0.45 (0.20, 0.78)	0.94 (0.90, 0.98)	0.52 (0.28, 0.76)

a Details of the SOM cluster analyses can be found in methods section.

b Index of toxic chemicals released by industrial facilities, converted to natural log units, transformed to z-scores and multiplied by −1.

c Average number of Superfund sites within a 2-mile radius, converted to natural log units, transformed to z-scores and multiplied by −1.

d Estimated percentage of homes at risk for lead exposure given lead-based paint in census tract of primary residential address.

e Proximity to major roads, in meters.

f Income disparity defined by Singh (2003) as the log of 100 × ratio of the number of households with <10,000 annual income to the number of households with >50,000 annual income.

g Percentage households without a car located further than a half-mile from the nearest supermarket, transformed to z-scores and multiplied by −1.

h Percentage impenetrable surface areas such as rooftops, roads or parking lots, transformed to z-scores and multiplied by −1.

i EPA Walkability Index.

## Data Availability

The data is publicly available for use upon request (authors do not own the data).
