# Supplementary material for: Association of social and environmental exposures at the neighborhood level with child brain volume
Source: Environ Int. Author manuscript; Available in PMC 2025 Jul 10. (PMC12242557; doi:10.1016/j.envint.2025.109576)
Supplement: 2 [file NIHMS2091160-supplement-2.docx]

**SUPPLEMENTAL TABLES AND FIGURES**

**Supplemental Table 1.**

Exposure definitions for neighborhood-level exposures

| **Exposure Mixture Component** | **Index/Domain** | **Definition** | **Year(s) Measured** |
| --- | --- | --- | --- |
| **Environmental Exposures** |  |  |  |
| PM_2.5_ (μg/m3) | Air Quality | Spatio-temporal model predictions  measured in μg/m3 at 1 km2  resolution | 2016 |
| NO_2_ (ppb) | Air Quality | Spatio-temporal model predictions  measured in ppb (parts per billion) at  1 km2 resolution | 2016 |
| O_3_ (ppb) | Air Quality | Spatio-temporal model predictions  measured in ppb (parts per billion) at  1 km2 resolution | 2016 |
| Industrial Pollutants | Child Opportunity Index 2.0 | Index of toxic chemicals released by industrial facilities, converted to natural log units, transformed to z-scores and multiplied by -1 | 2015 |
| Hazardous Waste Sites | Child Opportunity Index 2.0 | Average number of Superfund sites within a 2-mile radius, converted to natural log units, transformed to z-scores and multiplied by -1 | 2015 |
| Lead Risk | Lead Risk | Estimated percentage of homes at risk for lead exposure given lead-based paint in census tract of primary residential address | 2010-2014 |
| Proximity to Roadways | Air Quality | Proximity to major roads, in meters | NA |
| **Social Exposures** |  |  |  |
| Percent Single Parent Households | Area Deprivation Index | Percentage of single parent households | 2010-2014 |
| Percent Home Ownership | Area Deprivation Index | Percentage of owner-occupied households | 2010-2014 |
| % Less Than 9 Years Education | Area Deprivation Index | Percentage of population aged >=25 years with <9 years of education | 2010-2014 |
| % With at Least High School Diploma | Area Deprivation Index | Percentage of population aged >=25 years with at least a high school diploma | 2010-2014 |
| % White Collar Occupation | Area Deprivation Index | Percentage of employed persons aged >=16 years in white collar occupations | 2010-2014 |
| Median Family Income | Area Deprivation Index | Median family income | 2010-2014 |
| Income Disparity | Area Deprivation Index | Income disparity defined by Singh (2003) as the log of 100 x ratio of the number of households with <10000 annual income to the number of households with >50000 annual income | 2010-2014 |
| Median Home Value | Area Deprivation Index | Median home value | 2010-2014 |
| Median Gross Rent | Area Deprivation Index | Median gross rent | 2010-2014 |
| Median Monthly Mortgage | Area Deprivation Index | Median monthly mortgage | 2010-2014 |
| Crowding | Area Deprivation Index | Percentage of occupied housing units with >1 person per room (crowding) | 2010-2014 |
| % Unemployment | Area Deprivation Index | Percentage of civilian labor force population aged >=16 y unemployed (unemployment rate) | 2010-2014 |
| % Below Poverty Line | Area Deprivation Index | Percentage of families below the poverty level | 2010-2014 |
| % Below 138% Poverty Line | Area Deprivation Index | Percentage of population below 138% of the poverty threshold | 2010-2014 |
| % Households with No Car | Area Deprivation Index | Percentage of occupied housing units without a motor vehicle | 2010-2014 |
| % Poor Plumbing | Area Deprivation Index | Percentage of occupied housing units without complete plumbing (log) | 2010-2014 |
| Access to Food | Child Opportunity Index 2.0 | Percentage households without a car located further than a half-mile from the nearest supermarket, transformed to z-scores and multiplied by -1 | 2015 |
| Access to Green Space | Child Opportunity Index 2.0 | Percentage impenetrable surface areas such as rooftops, roads or parking lots, transformed to z-scores and multiplied by -1 | 2015 |
| Walkability | Child Opportunity Index 2.0 | EPA Walkability Index | 2015 |
| Total Crime | Crime | County level counts of arrests and  offences from Uniform Crime  Reporting Program Data | 2010-2012 |
| Percent Minority Population | Social Vulnerability Index | Percentage minority population (i.e., all but white, non-Hispanic) | 2014-2018 |
| Percent Non-English Speakers | Social Vulnerability Index | Percentage of persons at least 5 years old who speak English “less than well” | 2014-2018 |

**
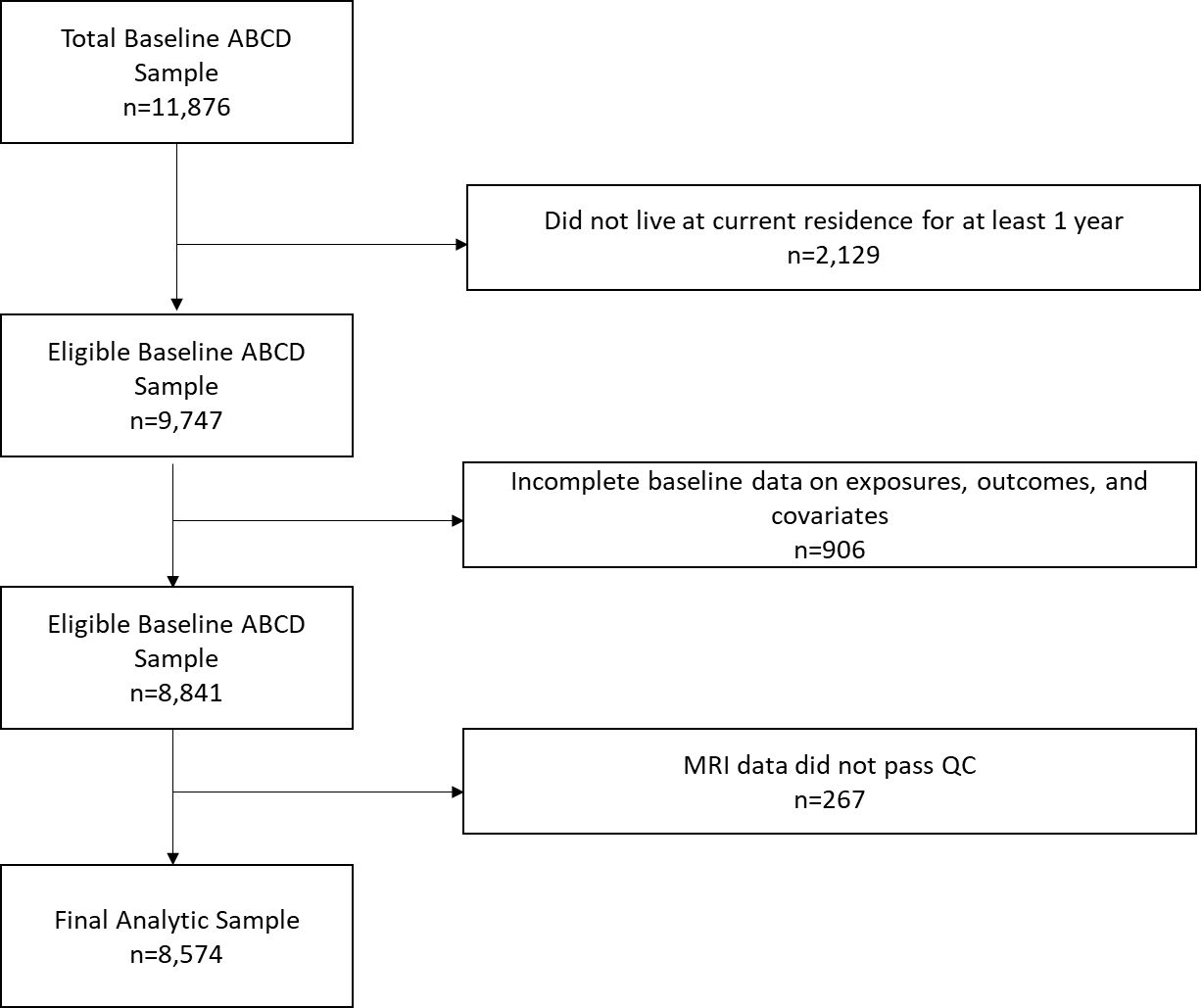
**

**Supplemental Figure 1.** Selection of participants from the Adolescent Brain Cognitive Development study for analytic sample in a study of neighborhood-level exposures and brain volume.

**Supplemental Table 2**. Description of Total Baseline Adolescent Brain Cognitive Development (ABCD) study sample, pre-exclusions (N=11,876) vs. post-exclusions.

| **Sample Characteristics** | Total Sample | Analytic sample |
| --- | --- | --- |
| Female Sex, n (%) | 5,614 (48%) | 4,108 (48%) |
| Age, median (IQR) | 9.92 (9.33, 10.50) | 9.92 (9.33, 10.50) |
| Race, n (%) |  |  |
| White | 7,522 (64%) | 5,710 (67%) |
| Black | 1,868 (16%) | 1,173 (14%) |
| American Indian or Alaskan Native | 62 (0.5%) | 40 (0.5%) |
| Asian | 243 (2.1%) | 196 (2.3%) |
| Pacific Islander | 15 (0.1%) | 10 (0.1%) |
| Some Other Race | 525 (4.5%) | 384 (4.5%) |
| Two or More Races | 1,467 (13%) | 1,061 (12%) |
| Missing Race | 171 (1.4%) | - |
| Hispanic Ethnicity, n (%) | 2,410 (21%) | 1,668 (19%) |
| Missing Ethnicity | 153 (1.3%) | - |
| Highest Level of Parent Education, n (%) |  |  |
| Less than college | 1,725 (15%) | 1,046 (12%) |
| Some College | 1,506 (13%) | 1,024 (12%) |
| Associate’s Degree | 1,571 (13%) | 1,102 (13%) |
| Bachelor’s Degree | 3,014 (25%) | 2,269 (26%) |
| Master’s Degree | 2,806 (24%) | 2,176 (25%) |
| Professional/Doctoral Degree | 1,237 (10%) | 957 (11%) |
| Both Parents Missing Education Level | 14 (0.12%) | - |
| Household Income, n (%) |  |  |
| Less than $50,000 | 4,473 (41%) | 3,015 (38%) |
| $50,000- $99,999 | 3,071 (28%) | 2,299 (29%) |
| $100,000+ | 3,312 (31%) | 2,590 (33%) |
| Missing income | 1,017 (8.6%) | 670 (8%) |

**Supplemental Table 3**. Median brain volume (in mm^3^) and IQR of examined brain regions by SOM cluster

|  |  | **SOM Cluster** | | | | |
| --- | --- | --- | --- | --- | --- | --- |
|  | **Total** | **1** | **2** | **3** | **4** | **5** |
| N (%) | 8,574 (100) | 2,642 (30.81) | 2,686 (31.33) | 1,561 (18.21) | 760 (8.86) | 925 (10.79) |
| Intracranial Volume | 1,491,630 (1,396,632, 1,590,978) | 1,505,200 (1,419,038, 1,605,464) | 1,508,419 (1,406,443, 1,603,842) | 1,491,135 (1,403,542, 1,589,219) | 1,439,340 (1,343,397, 1,540,660) | 1,444,906 (1,353,343, 1,542,327) |
| Left Amygdala | 1,705 (1,555, 1,869) | 1,733 (1,572, 1,897) | 1,718 (1,565, 1,885) | 1,709 (1,572, 1,873) | 1,668 (1,522, 1,813) | 1,620 (1,492, 1,779) |
| Right Amygdala | 1,822 (1,680, 1,970) | 1,836 (1,703, 2,000) | 1,834 (1,686, 1,979) | 1,828 (1,696, 1,969) | 1,796 (1,651, 1,935) | 1,742 (1,617, 1,897) |
| Left Hippocampus | 4,017 (3,759, 4,290) | 4,068 (3,809, 4,336) | 4,052 (3,787, 4,319) | 4,004 (3,768, 4,276) | 3,937 (3,677, 4,190) | 3,844 (3,622, 4,103) |
| Right Hippocampus | 4,143 (3,869, 4,426) | 4,196 (3,913, 4,478) | 4,171 (3,910, 4,464) | 4,141 (3,882, 4,411) | 4,076 (3,821, 4,349) | 3,948 (3,718, 4,228) |
| Left Superior Frontal Gyrus | 29,610 (27,327, 32,053) | 29,971 (27,789, 32,498) | 29,877 (27,527, 32,436) | 29,525 (27,205, 31,712) | 28,676 (26,530, 31,169) | 28,517 (26,333, 30,924) |
| Right Superior Frontal Gyrus | 28,250 (26,061, 30,674) | 28,695 (26,522, 31,207) | 28,543 (26,316, 30,964) | 28,110 (26,031, 30,380) | 27,352 (25,243, 29,488) | 27,227 (25,056, 29,544) |
| Left Caudal Anterior Cingulate Cortex | 2,092 (1,761, 2,504) | 2,147 (1,792, 2,566) | 2,108 (1,802, 2,519) | 2,089 (1,739, 2,496) | 1,966 (1,668, 2,342) | 1,965 (1,662, 2,388) |
| Right Caudal Anterior Cingulate Cortex | 2,432 (2,061, 2,834) | 2,464 (2,100, 2,875) | 2,440 (2,073, 2,846) | 2,407 (2,074, 2,818) | 2,420 (1,995, 2,791) | 2,341 (1,961, 2,727) |
| Left Caudal Middle Frontal Gyrus | 8,212 (7,234, 9,252) | 8,396 (7,425, 9,419) | 8,317 (7,372, 9,410) | 8,130 (7,154, 9,174) | 7,814 (6,865, 8,799) | 7,837 (6,870, 8,892) |
| Right Caudal Middle Frontal Gyrus | 7,869 (6,902, 8,914) | 7,978 (7,070, 9,038) | 7,979 (7,026, 9,003) | 7,832 (6,845, 8,864) | 7,563 (6,589, 8,592) | 7,433 (6,500, 8,449) |
| Left Lateral Orbitofrontal Cortex | 9,514 (8,798, 10,266) | 9,636 (8,935, 10,403) | 9,604 (8,864, 10,356) | 9,514 (8,851, 10,199) | 9,320 (8,672, 9,969) | 9,013 (8,346, 9,855) |
| Right Lateral Orbitofrontal Cortex | 9,027 (8,310, 9,780) | 9,169 (8,476, 9,911) | 9,095 (8,386, 9,840) | 8,918 (8,257, 9,623) | 8,882 (8,278, 9,586) | 8,625 (7,838, 9,471) |
| Left Medial Orbitofrontal Cortex | 5,925 (5,397, 6,494) | 6,022 (5,464, 6,560) | 5,957 (5,409, 6,530) | 5,862 (5,325, 6,414) | 5,917 (5,448, 6,418) | 5,747 (5,275, 6,360) |
| Right Medial Orbitofrontal Cortex | 6,722 (6,195, 7,268) | 6,796 (6,276, 7,338) | 6,770 (6,240, 7,332) | 6,696 (6,187, 7,244) | 6,632 (6,114, 7,131) | 6,511 (5,992, 7,085) |
| Left Pars Opercularis | 5,964 (5,333, 6,681) | 6,059 (5,410, 6,806) | 6,036 (5,404, 6,699) | 5,970 (5,326, 6,656) | 5,752 (5,164, 6,495) | 5,704 (5,101, 6,407) |
| Right Pars Opercularis | 4,934 (4,445, 5,502) | 5,041 (4,521, 5,613) | 4,963 (4,482, 5,553) | 4,892 (4,416, 5,429) | 4,748 (4,339, 5,299) | 4,777 (4,262, 5,347) |
| Left Pars Triangularis | 4,964 (4,435, 5,559) | 4,979 (4,454, 5,627) | 5,014 (4,457, 5,586) | 4,942 (4,452, 5,533) | 4,913 (4,370, 5,456) | 4,828 (4,344, 5,446) |
| Right Pars Triangularis | 5,771 (5,169, 6,440) | 5,845 (5,206, 6,534) | 5,792 (5,189, 6,462) | 5,725 (5,118, 6,372) | 5,681 (5,166, 6,353) | 5,697 (5,118, 6,319) |
| Left Pars Orbitalis | 3,203 (2,918, 3,493) | 3,256 (2,977, 3,537) | 3,222 (2,931, 3,511) | 3,217 (2,937, 3,498) | 3,072 (2,813, 3,364) | 3,053 (2,782, 3,382) |
| Right Pars Orbitalis | 3,771 (3,448, 4,120) | 3,840 (3,513, 4,175) | 3,791 (3,473, 4,148) | 3,768 (3,441, 4,124) | 3,662 (3,345, 3,978) | 3,630 (3,290, 3,971) |
| Left Rostral Anterior Cingulate Cortex | 3,181 (2,769, 3,607) | 3,226 (2,810, 3,649) | 3,253 (2,847, 3,663) | 3,123 (2,727, 3,564) | 3,078 (2,625, 3,496) | 3,045 (2,623, 3,483) |
| Right Rostral Anterior Cingulate Cortex | 2,358 (2,028, 2,710) | 2,381 (2,068, 2,726) | 2,399 (2,067, 2,749) | 2,329 (2,003, 2,700) | 2,305 (1,949, 2,643) | 2,262 (1,925, 2,613) |
| Left Rostral Middle Frontal Gyrus | 21,006 (19,227, 22,892) | 21,343 (19,562, 23,219) | 21,202 (19,488, 23,023) | 20,871 (19,137, 22,696) | 20,021 (18,281, 21,821) | 20,265 (18,670, 22,390) |
| Right Rostral Middle Frontal Gyrus | 21,696 (19,804, 23,693) | 22,095 (20,225, 23,982) | 21,961 (19,974, 23,943) | 21,551 (19,639, 23,521) | 20,613 (18,911, 22,638) | 21,181 (19,150, 23,129) |
| Left Frontal Pole | 1,337 (1,214, 1,475) | 1,354 (1,233, 1,493) | 1,344 (1,222, 1,477) | 1,353 (1,216, 1,486) | 1,286 (1,160, 1,418) | 1,301 (1,181, 1,434) |
| Right Frontal Pole | 1,642 (1,488, 1,802) | 1,671 (1,509, 1,830) | 1,651 (1,494, 1,807) | 1,649 (1,503, 1,808) | 1,562 (1,413, 1,750) | 1,582 (1,456, 1,735) |
| Left Insula | 7,783 (7,208, 8,383) | 7,894 (7,308, 8,480) | 7,856 (7,310, 8,442) | 7,736 (7,108, 8,292) | 7,556 (7,023, 8,141) | 7,572 (6,972, 8,204) |
| Right Insula | 7,496 (6,939, 8,084) | 7,589 (7,038, 8,174) | 7,589 (7,033, 8,158) | 7,386 (6,868, 8,011) | 7,266 (6,766, 7,840) | 7,303 (6,784, 7,901) |

**
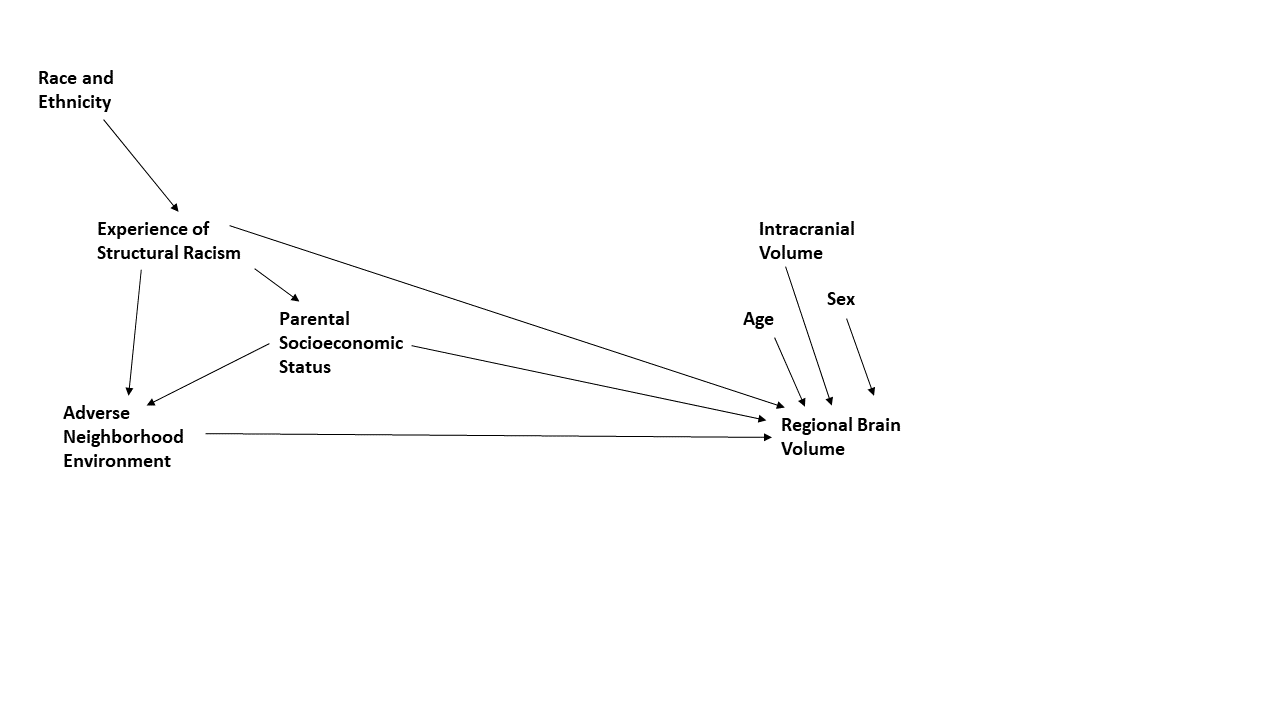
Supplemental Figure 2.** Directed Acyclic Graph (DAG) depicting causal relationships between exposure mixture, outcomes, and covariates included in models.

**
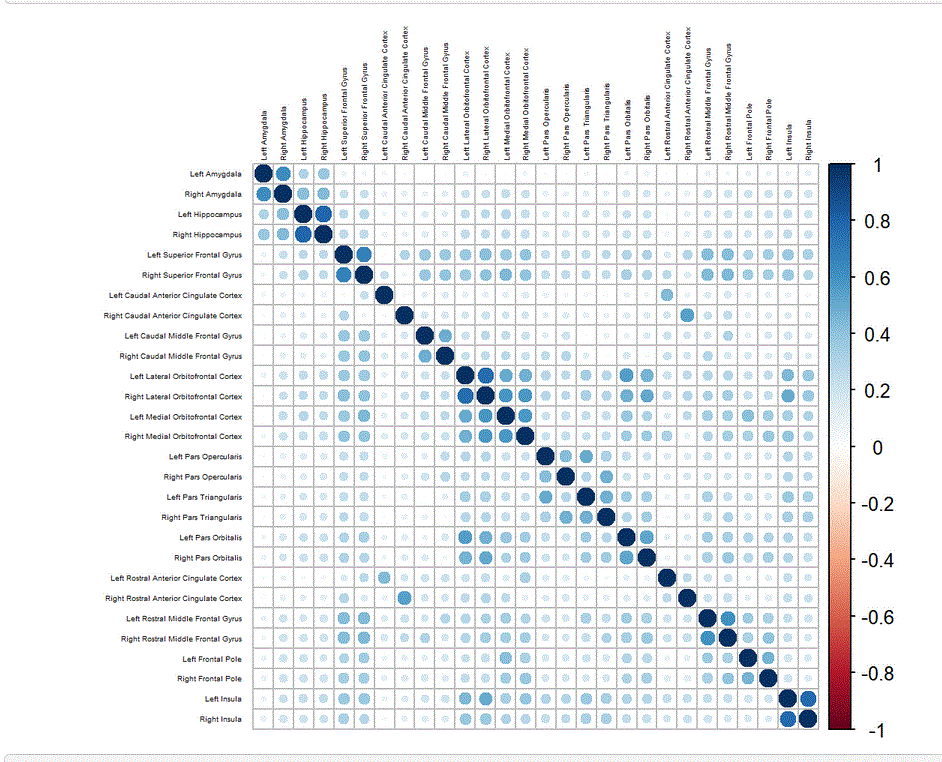
**

**Supplemental Figure 3.** Correlation matrix of all included outcomes divide by intracranial volume. Darker blue shades represent greater positive correlations, while darker red shades represent greater negative correlations.

**
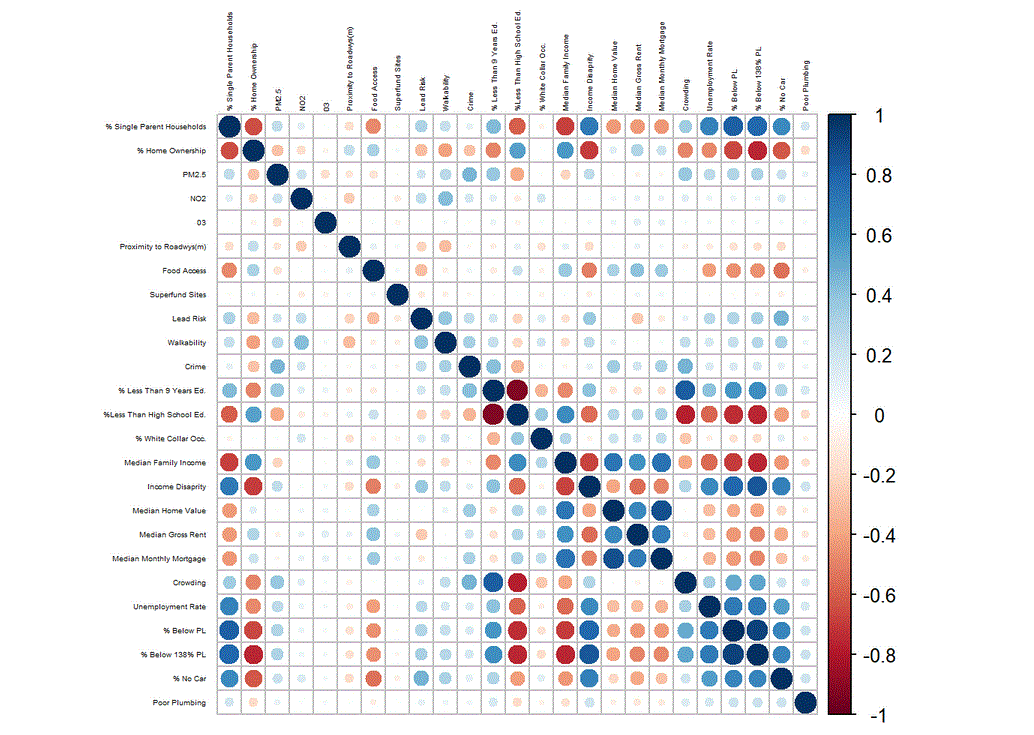
**

**Supplemental Figure 4.** Correlation matrix of all included exposures. Darker blue shades represent greater positive correlations, while darker red shades represent greater negative correlations.

**Supplemental Table 4.** Associations between SOM cluster and volume of 16 lateralized brain regions (N=8,574)

|  | **SOM Cluster** | |  | |  | |  | |
| --- | --- | --- | --- | --- | --- | --- | --- | --- |
|  | **2** | | **3** | | **4** | | **5** | |
| **Region** | Beta | 95% CI | Beta | 95% CI | Beta | 95% CI | Beta | 95% CI |
| **Left Amygdala**  Base Model* | -0.05 | -0.10  -0.01 | -0.06 | -0.11  0.00 | -0.03 | -0.11  0.04 | -0.25 | -0.32  -0.19 |
| Fully Adjusted** | -0.02 | -0.07, 0.03 | -0.02 | -0.07, 0.04 | 0.02 | -0.06, 0.10 | -0.07 | -0.14, 0.01 |
| **Right Amygdala**  Base Model | -0.03 | -0.08  0.02 | -0.02 | -0.08  0.03 | 0.02 | -0.05  0.09 | -0.18 | -0.25  -0.12 |
| Fully Adjusted | 0.00 | -0.05  0.05 | 0.01 | -0.05  0.07 | 0.04 | -0.04  0.13 | -0.04 | -0.12  0.04 |
| **Left Hippocampus**  Base Model | 0.00 | -0.05  0.04 | -0.03 | -0.09  0.02 | -0.08 | -0.15  -0.01 | -0.23 | -0.29  -0.16 |
| Fully Adjusted | 0.04 | -0.01  0.08 | 0.02 | -0.04  0.07 | 0.02 | -0.06  0.10 | -0.03 | \| -0.11 \| \| --- \| \| 0.04 \| |
| **Right Hippocampus**  Base Model | -0.01 | -0.06  0.04 | -0.04 | -0.09  0.02 | -0.05 | -0.12  0.02 | -0.26 | -0.33  -0.19 |
| Fully Adjusted | 0.02 | -0.03  0.07 | 0.00 | -0.05  0.06 | 0.00 | -0.08  0.08 | -0.08 | -0.16  0.00 |
| **Left Superior Frontal Gyrus**  Base Model | -0.03 | -0.08  0.01 | 0.00 | -0.06  0.05 | -0.15 | -0.22  -0.08 | -0.07 | -0.13  -0.01 |
| Fully Adjusted | -0.01 | -0.06  0.03 | 0.03 | -0.02  0.08 | -0.01 | -0.09  0.06 | 0.01 | -0.06  0.08 |
| **Right Superior Frontal Gyrus**  Base Model | -0.02 | -0.06  0.03 | -0.02 | -0.07  0.03 | -0.19 | -0.26  -0.12 | -0.07 | -0.13  -0.01 |
| Fully Adjusted | 0.01 | -0.04  0.05 | 0.02 | -0.03  0.08 | -0.03 | -0.10  0.05 | 0.03 | -0.04  0.10 |
| **Left Caudal Anterior Cingulate Cortex**  Base Model | -0.04 | -0.10  0.01 | -0.04 | -0.10  0.02 | -0.15 | -0.23  -0.07 | -0.10 | -0.17  -0.02 |
| Fully Adjusted | -0.03 | -0.08  0.03 | -0.01 | -0.07  0.06 | -0.08 | -0.18  0.01 | -0.02 | -0.11  0.07 |
| **Right Caudal Anterior Cingulate Cortex**  Base Model | 0.00 | -0.06  0.05 | 0.06 | 0.00  0.12 | 0.01 | -0.07  0.09 | -0.02 | -0.10  0.05 |
| Fully Adjusted | 0.01 | -0.04  0.07 | 0.08 | 0.02  0.15 | 0.02 | -0.08  0.11 | 0.07 | -0.02  0.16 |
| **Left Caudal Middle Frontal Gyrus**  Base Model | -0.02 | -0.07  0.03 | 0.02 | -0.04  0.08 | -0.21 | -0.29  -0.14 | -0.09 | -0.16  -0.02 |
| Fully Adjusted | 0.00 | -0.05  0.05 | 0.05 | -0.01  0.11 | -0.08 | -0.16  0.01 | -0.01 | -0.09  0.07 |
| **Right Caudal Middle Frontal Gyrus**  Base Model | -0.02 | -0.07  0.03 | 0.07 | 0.01  0.13 | -0.12 | -0.19  -0.04 | -0.11 | -0.18  -0.04 |
| Fully Adjusted | 0.01 | -0.04  0.06 | 0.11 | 0.05  0.17 | 0.01 | -0.08  0.10 | 0.01 | -0.08  0.09 |
| **Left Lateral Orbitofrontal Cortex**  Base Model | -0.05 | -0.09  0.00 | -0.03 | -0.09  0.02 | -0.08 | -0.15  -0.02 | -0.24 | -0.30  -0.18 |
| Fully Adjusted | 0.00 | -0.04  0.04 | 0.00 | -0.05  0.06 | 0.00 | -0.08  0.07 | 0.00 | \| -0.07 \| \| --- \| \| 0.07 \| |
| **Right Lateral Orbitofrontal Cortex**  Base Model | -0.05 | -0.10  -0.01 | -0.03 | -0.08  0.02 | -0.11 | -0.18  -0.05 | -0.16 | -0.22  -0.10 |
| Fully Adjusted | -0.02 | -0.07  0.02 | 0.00 | -0.05  0.05 | -0.05 | -0.12  0.03 | -0.01 | -0.08  0.06 |
| **Left Medial Orbitofrontal Cortex**  Base Model | 0.00 | -0.04  0.04 | -0.02 | -0.07  0.03 | -0.01 | -0.08  0.05 | 0.05 | -0.01  0.11 |
| Fully Adjusted | 0.00 | -0.04  0.04 | -0.01 | -0.06  0.04 | 0.01 | -0.06  0.09 | 0.04 | -0.03  0.11 |
| **Right Medial Orbitofrontal Cortex**  Base Model | 0.00 | -0.04  0.05 | 0.00 | -0.05  0.05 | -0.05 | -0.12  0.01 | 0.00 | -0.06  0.06 |
| Fully Adjusted | 0.02 | -0.03  0.06 | 0.02 | -0.03  0.08 | 0.01 | -0.07  0.09 | 0.05 | -0.02  0.13 |
| **Left Pars Opercularis**  Base Model | -0.05 | -0.10  0.00 | -0.02 | -0.08  0.04 | -0.13 | -0.22  -0.05 | -0.14 | -0.22  -0.07 |
| Fully Adjusted | -0.03 | -0.08  0.03 | 0.00 | -0.06  0.07 | -0.04 | -0.13  0.05 | -0.04 | -0.12  0.05 |
| **Right Pars Opercularis**  Base Model | -0.07 | -0.12  -0.01 | -0.04 | -0.11  0.02 | -0.14 | -0.22  -0.06 | -0.08 | -0.16  -0.01 |
| Fully Adjusted | -0.05 | -0.10  0.01 | -0.02 | -0.09  0.04 | -0.10 | -0.19  -0.01 | 0.02 | -0.06  0.11 |
| **Left Pars Triangularis**  Base Model | -0.02 | -0.07  0.04 | -0.01 | -0.07  0.06 | -0.03 | -0.11  0.06 | -0.02 | -0.09  0.06 |
| Fully Adjusted | -0.01 | -0.07  0.05 | 0.01 | -0.06  0.07 | 0.02 | -0.07  0.12 | 0.02 | -0.07  0.11 |
| **Right Pars Triangularis**  Base Model | -0.05 | -0.10  0.01 | -0.04 | -0.11  0.02 | -0.04 | -0.12  0.04 | 0.05 | -0.02  0.13 |
| Fully Adjusted | -0.06 | -0.11  0.00 | -0.05 | -0.12  0.02 | -0.07 | -0.17  0.02 | 0.03 | -0.06  0.12 |
| **Left Pars Orbitalis**  Base Model | -0.10 | -0.15  -0.05 | -0.02 | -0.08  0.04 | -0.24 | -0.32  -0.17 | -0.21 | -0.28  -0.14 |
| Fully Adjusted | -0.07 | -0.12  -0.02 | 0.01 | -0.05  0.07 | -0.14 | -0.23  -0.06 | -0.06 | -0.14  0.02 |
| **Right Pars Orbitalis**  Base Model | -0.09 | -0.14  -0.04 | -0.01 | -0.07  0.05 | -0.19 | -0.27  -0.11 | -0.19 | -0.26  -0.12 |
| Fully Adjusted | -0.07 | -0.12  -0.01 | 0.02 | -0.05  0.08 | -0.11 | -0.20  -0.02 | -0.08 | -0.17  0.00 |
| **Left Rostral Anterior Cingulate Cortex**  Base Model | 0.01 | -0.04  0.06 | -0.03 | -0.08  0.03 | -0.04 | -0.11  0.03 | -0.03 | -0.10  0.04 |
| Fully Adjusted | 0.04 | -0.01  0.09 | 0.01 | -0.05  0.07 | 0.05 | -0.04  0.13 | 0.07 | -0.01  0.15 |
| **Right Rostral Anterior Cingulate Cortex**  Base Model | 0.00 | -0.05  0.05 | 0.01 | -0.05  0.07 | -0.04 | -0.12  0.03 | -0.09 | -0.16  -0.02 |
| Fully Adjusted | 0.02 | -0.03  0.07 | 0.03 | -0.03  0.09 | -0.01 | -0.09  0.08 | 0.02 | -0.06  0.10 |
| **Left Rostral Middle Frontal Gyrus**  Base Model | -0.04 | -0.09  0.01 | -0.05 | -0.10  0.01 | -0.23 | -0.30  -0.16 | -0.05 | -0.12  0.01 |
| Fully Adjusted | -0.02 | -0.07  0.03 | 0.00 | -0.06  0.06 | -0.07 | -0.15  0.01 | -0.01 | -0.08  0.07 |
| **Right Rostral Middle Frontal Gyrus**  Base Model | -0.04 | -0.09  0.01 | -0.06 | -0.11  0.00 | -0.25 | -0.32  -0.18 | -0.05 | -0.12  0.01 |
| Fully Adjusted | -0.03 | -0.07  0.02 | -0.02 | -0.07  0.04 | -0.10 | -0.18  -0.02 | -0.02 | -0.10  0.06 |
| **Left Frontal Pole**  Base Model | -0.04 | -0.09  0.01 | -0.05 | -0.10  0.01 | -0.23 | -0.30  -0.16 | -0.05 | -0.12  0.01 |
| Fully Adjusted | -0.02 | -0.07  0.03 | 0.00 | -0.06  0.06 | -0.07 | -0.15  0.01 | -0.01 | -0.08  0.07 |
| **Right Frontal Pole**  Base Model | -0.04 | -0.09  0.01 | -0.06 | -0.11  0.00 | -0.25 | -0.32  -0.18 | -0.05 | -0.12  0.01 |
| Fully Adjusted | -0.03 | -0.07  0.02 | -0.02 | -0.07  0.04 | -0.10 | -0.18  -0.02 | -0.02 | -0.10  0.06 |
| **Left Insula**  Base Model | -0.04 | -0.09  0.00 | -0.04 | -0.09  0.02 | -0.14 | -0.21  -0.07 | -0.10 | -0.16  -0.03 |
| Fully Adjusted | -0.02 | -0.07  0.02 | 0.00 | -0.06  0.05 | -0.06 | -0.14  0.02 | -0.01 | -0.09  0.06 |
| **Right Insula**  Base Model | -0.03 | -0.07  0.02 | -0.04 | -0.09  0.02 | -0.12 | -0.19  -0.05 | -0.06 | -0.13  0.00 |
| Fully Adjusted | -0.01 | -0.06  0.03 | -0.02 | -0.07  0.04 | -0.06 | -0.14  0.01 | -0.01 | -0.08  0.07 |

* Adjusting for family relatedness as a random effect, and intracranial volume, child age, and sex as fixed effects.

** Adjusting for family relatedness as a random effect, and intracranial volume, child age, sex, race, ethnicity, and parents’ highest level of education as fixed effects.

**Supplemental Table 5.** Associations between SOM cluster and volume of 16 lateralized brain regions when excluding all children on psychiatric medication (N=7,829)

|  | **SOM Cluster** | |  | |  | |  | |
| --- | --- | --- | --- | --- | --- | --- | --- | --- |
|  | **2** | | **3** | | **4** | | **5** | |
| **Region** | Beta | 95% CI | Beta | 95% CI | Beta | 95% CI | Beta | 95% CI |
| **Left Amygdala** | -0.01 | -0.07  0.04 | -0.02 | -0.08  0.04 | 0.03 | -0.06  0.12 | -0.07 | -0.15  0.01 |
| **Right Amygdala** | 0.01 | -0.04  0.06 | 0.00 | -0.06  0.06 | 0.07 | -0.01  0.16 | -0.02 | -0.11  0.06 |
| **Left Hippocampus** | 0.04 | -0.01  0.09 | 0.01 | -0.05  0.07 | 0.04 | -0.05  0.12 | -0.02 | -0.10  0.05 |
| **Right Hippocampus** | 0.03 | -0.02  0.08 | 0.00 | -0.06  0.06 | 0.03 | -0.06  0.11 | -0.07 | -0.15  0.01 |
| **Left Superior Frontal Gyrus** | -0.01 | -0.05  0.04 | 0.04 | -0.01  0.10 | 0.00 | -0.08  0.08 | 0.02 | -0.06  0.09 |
| **Right Superior Frontal Gyrus** | 0.00 | -0.04  0.05 | 0.02 | -0.03  0.08 | -0.03 | -0.11  0.05 | 0.02 | -0.06  0.09 |
| **Left Caudal Anterior Cingulate Cortex** | -0.04 | -0.10  0.02 | -0.02 | -0.09  0.05 | -0.08 | -0.18  0.02 | -0.03 | -0.12  0.07 |
| **Right Caudal Anterior Cingulate Cortex** | 0.02 | -0.04  0.08 | 0.10 | 0.03  0.17 | 0.04 | -0.06  0.13 | 0.07 | -0.03  0.16 |
| **Left Caudal Middle Frontal Gyrus** | 0.01 | -0.04  0.06 | 0.07 | 0.00  0.13 | -0.08 | -0.17  0.00 | 0.00 | -0.08  0.09 |
| **Right Caudal Middle Frontal Gyrus** | 0.02 | -0.03  0.07 | 0.13 | 0.07  0.19 | 0.02 | -0.07  0.11 | 0.02 | -0.07  0.11 |
| **Left Lateral Orbitofrontal Cortex** | 0.02 | -0.03  0.07 | 0.02 | -0.04  0.07 | 0.02 | -0.06  0.09 | 0.01 | -0.06  0.09 |
| **Right Lateral Orbitofrontal Cortex** | -0.01 | -0.05  0.04 | 0.00 | -0.05  0.06 | -0.03 | -0.10  0.05 | 0.00 | -0.07  0.08 |
| **Left Medial Orbitofrontal Cortex** | 0.02 | -0.03  0.06 | 0.00 | -0.05  0.06 | 0.04 | -0.04  0.12 | 0.05 | -0.02  0.12 |
| **Right Medial Orbitofrontal Cortex** | 0.03 | -0.02  0.08 | 0.03 | -0.03  0.08 | 0.03 | -0.05  0.11 | 0.06 | -0.02  0.13 |
| **Left Pars Opercularis** | -0.02 | -0.08  0.04 | 0.00 | -0.06  0.07 | -0.04 | -0.14  0.06 | -0.03 | -0.12  0.06 |
| **Right Pars Opercularis** | -0.04 | -0.10  0.01 | -0.02 | -0.08  0.05 | -0.08 | -0.18  0.01 | 0.03 | -0.07  0.12 |
| **Left Pars Triangularis** | -0.02 | -0.07  0.04 | -0.01 | -0.08  0.06 | 0.00 | -0.10  0.10 | 0.00 | -0.09  0.09 |
| **Right Pars Triangularis** | -0.06 | -0.12  0.00 | -0.06 | -0.13  0.01 | -0.08 | -0.18  0.02 | 0.01 | -0.08  0.10 |
| **Left Pars Orbitalis** | -0.06 | -0.11  -0.01 | 0.02 | -0.04  0.08 | -0.14 | -0.22  -0.05 | -0.05 | -0.14  0.03 |
| **Right Pars Orbitalis** | -0.07 | -0.13  -0.02 | 0.03 | -0.04  0.09 | -0.11 | -0.20  -0.02 | -0.08 | -0.17  0.01 |
| **Left Rostral Anterior Cingulate Cortex** | 0.03 | -0.02  0.08 | 0.01 | -0.06  0.07 | 0.05 | -0.04  0.14 | 0.06 | -0.02  0.15 |
| **Right Rostral Anterior Cingulate Cortex** | 0.04 | -0.01  0.09 | 0.06 | -0.01  0.12 | 0.01 | -0.08  0.10 | 0.02 | -0.07  0.10 |
| **Left Rostral Middle Frontal Gyrus** | -0.03 | -0.08  0.02 | 0.01 | -0.05  0.07 | -0.07 | -0.15  0.02 | 0.00 | -0.08  0.08 |
| **Right Rostral Middle Frontal Gyrus** | -0.02 | -0.07  0.03 | -0.01 | -0.07  0.05 | -0.10 | -0.19  -0.02 | -0.02 | -0.10  0.06 |
| **Left Frontal Pole** | -0.03 | -0.08  0.02 | 0.01 | -0.05  0.07 | -0.07 | -0.15  0.02 | 0.00 | -0.08  0.08 |
| **Right Frontal Pole** | -0.02 | -0.07  0.03 | -0.01 | -0.07  0.05 | -0.10 | -0.19  -0.02 | -0.02 | -0.10  0.06 |
| **Left Insula** | -0.02 | -0.07  0.03 | 0.00 | -0.06  0.06 | -0.05 | -0.14  0.03 | 0.00 | -0.07  0.08 |
| **Right Insula** | -0.01 | -0.05  0.04 | -0.01 | -0.07  0.05 | -0.06 | -0.15  0.02 | 0.01 | -0.07  0.09 |

*Adjusting for family relatedness as a random effect, and intracranial volume, child age, and sex as fixed effects.

** Adjusting for family relatedness as a random effect, and intracranial volume, child age, sex, race, ethnicity, and parents’ highest level of education as fixed effects.

**Supplemental Table 6.** Associations between SOM cluster and volume of 16 lateralized brain regions, with additional adjustment for household income to poverty ratio (N=7,799)

|  | **SOM Cluster** | |  | |  | |  | |
| --- | --- | --- | --- | --- | --- | --- | --- | --- |
|  | **2** | | **3** | | **4** | | **5** | |
| **Region** | Beta | 95% CI | Beta | 95% CI | Beta | 95% CI | Beta | 95% CI |
| **Left Amygdala** | -0.02 | -0.07  0.03 | -0.01 | -0.08  0.05 | 0.03 | -0.06  0.12 | -0.09 | -0.17  0.00 |
| **Right Amygdala** | 0.00 | -0.05  0.05 | 0.02 | -0.04  0.08 | 0.04 | -0.05  0.13 | -0.06 | -0.15  0.02 |
| **Left Hippocampus** | 0.04 | 0.00  0.09 | 0.01 | -0.04  0.07 | 0.00 | -0.08  0.09 | -0.05 | -0.13  0.03 |
| **Right Hippocampus** | 0.03 | -0.02  0.08 | 0.01 | -0.05  0.07 | -0.02 | -0.11  0.07 | -0.11 | -0.19  -0.03 |
| **Left Superior Frontal Gyrus** | -0.01 | -0.06  0.03 | 0.03 | -0.02  0.09 | -0.04 | -0.12  0.04 | -0.01 | -0.09  0.07 |
| **Right Superior Frontal Gyrus** | 0.01 | -0.03  0.06 | 0.03 | -0.02  0.09 | -0.04 | -0.12  0.04 | 0.01 | -0.06  0.09 |
| **Left Caudal Anterior Cingulate Cortex** | -0.04 | -0.09  0.02 | 0.00 | -0.07  0.07 | -0.06 | -0.16  0.04 | -0.03 | -0.13  0.06 |
| **Right Caudal Anterior Cingulate Cortex** | 0.00 | -0.05  0.06 | 0.08 | 0.01  0.15 | -0.01 | -0.11  0.09 | 0.07 | -0.03  0.16 |
| **Left Caudal Middle Frontal Gyrus** | 0.00 | -0.06  0.05 | 0.05 | -0.01  0.11 | -0.10 | -0.19  0.00 | -0.03 | -0.12  0.06 |
| **Right Caudal Middle Frontal Gyrus** | 0.00 | -0.05  0.05 | 0.09 | 0.03  0.16 | -0.03 | -0.12  0.07 | -0.01 | -0.09  0.08 |
| **Left Lateral Orbitofrontal Cortex** | 0.00 | -0.05  0.04 | 0.00 | -0.05  0.05 | 0.01 | -0.08  0.09 | 0.01 | -0.07  0.08 |
| **Right Lateral Orbitofrontal Cortex** | -0.03 | -0.07  0.02 | -0.01 | -0.06  0.05 | -0.05 | -0.13  0.03 | 0.00 | -0.08  0.07 |
| **Left Medial Orbitofrontal Cortex** | 0.00 | -0.04  0.05 | -0.02 | -0.07  0.04 | 0.00 | -0.08  0.08 | 0.05 | -0.03  0.12 |
| **Right Medial Orbitofrontal Cortex** | 0.01 | -0.04  0.06 | 0.02 | -0.04  0.07 | 0.01 | -0.07  0.10 | 0.06 | -0.02  0.14 |
| **Left Pars Opercularis** | -0.02 | -0.08  0.03 | 0.02 | -0.04  0.09 | -0.04 | -0.14  0.06 | -0.04 | -0.13  0.06 |
| **Right Pars Opercularis** | -0.05 | -0.11  0.01 | -0.03 | -0.09  0.04 | -0.12 | -0.22  -0.02 | 0.01 | -0.09  0.10 |
| **Left Pars Triangularis** | -0.01 | -0.06  0.05 | 0.01 | -0.06  0.08 | 0.01 | -0.09  0.11 | 0.03 | -0.07  0.12 |
| **Right Pars Triangularis** | -0.06 | -0.11  0.00 | -0.06 | -0.13  0.01 | -0.09 | -0.19  0.01 | 0.01 | -0.08  0.11 |
| **Left Pars Orbitalis** | -0.07 | -0.12  -0.01 | 0.01 | -0.05  0.08 | -0.12 | -0.22  -0.03 | -0.05 | -0.14  0.04 |
| **Right Pars Orbitalis** | -0.08 | -0.13  -0.02 | 0.02 | -0.04  0.08 | -0.16 | -0.25  -0.06 | -0.07 | -0.15  0.02 |
| **Left Rostral Anterior Cingulate Cortex** | 0.03 | -0.02  0.08 | 0.01 | -0.05  0.08 | 0.06 | -0.03  0.15 | 0.06 | -0.02  0.15 |
| **Right Rostral Anterior Cingulate Cortex** | 0.02 | -0.03  0.08 | 0.05 | -0.01  0.11 | -0.01 | -0.10  0.09 | 0.01 | -0.07  0.10 |
| **Left Rostral Middle Frontal Gyrus** | -0.02 | -0.06  0.03 | 0.01 | -0.05  0.07 | -0.07 | -0.16  0.02 | -0.04 | -0.12  0.04 |
| **Right Rostral Middle Frontal Gyrus** | -0.02 | -0.07  0.03 | -0.01 | -0.07  0.05 | -0.10 | -0.19  -0.02 | -0.04 | -0.13  0.04 |
| **Left Frontal Pole** | -0.02 | -0.06  0.03 | 0.01 | -0.05  0.07 | -0.07 | -0.16  0.02 | -0.04 | -0.12  0.04 |
| **Right Frontal Pole** | -0.02 | -0.07  0.03 | -0.01 | -0.07  0.05 | -0.10 | -0.19  -0.02 | -0.04 | -0.13  0.04 |
| **Left Insula** | -0.02 | -0.07  0.02 | 0.01 | -0.05  0.07 | -0.07 | -0.16  0.01 | -0.01 | -0.09  0.07 |
| **Right Insula** | -0.01 | -0.06  0.04 | -0.01 | -0.06  0.05 | -0.05 | -0.14  0.03 | -0.01 | -0.09  0.07 |

*Adjusting for family relatedness as a random effect, and intracranial volume, child age, and sex as fixed effects.

**Adjusting for family relatedness as a random effect, and intracranial volume, child age, sex, race, ethnicity, parents’ highest level of education, and household income to poverty ratio as fixed effects.

**Supplemental Table 7.** Associations between SOM cluster and right caudal middle frontal gyrus volume, when restricting sample to various durations residing at current residence

|  |  | **SOM Cluster** | | |  | |  | |  | | |
| --- | --- | --- | --- | --- | --- | --- | --- | --- | --- | --- | --- |
|  | **Sample Size** | **2** | | | **3** | | **4** | | **5** | | |
| **Duration** |  | Beta | 95% CI | Beta | | 95% CI | Beta | 95% CI | | Beta | 95% CI |
| **1+ Years**  **(main analysis)** | 8,574 | 0.01 | -0.04  0.06 | 0.11 | | 0.05  0.17 | 0.01 | -0.08  0.10 | | 0.01 | -0.08  0.09 |
| **2+ Years** | 5,247 | 0.03 | -0.03 0.10 | 0.12 | | 0.04 0.12 | 0.01 | -0.10 0.12 | | 0.02 | -0.09, 0.12 |
| **4+ Years** | 3,591 | 0.008 | -0.07 0.09 | 0.08 | | -0.01 0.17 | -0.04 | -0.18 0.09 | | -0.04 | -0.17 0.10 |
| **8+ Years** | 1,324 | -0.04 | -0.16 0.09 | 0.15 | | -0.009 0.30 | 0.09 | -0.13 0.31 | | -0.09 | -0.31 0.13 |

All models adjust for family relatedness as a random effect, and intracranial volume, child age, sex, race, ethnicity, parents’ highest level of education, and household income to poverty ratio as fixed effects.

**Supplemental Table 8.** Variance Inflation Factors for Exposure and Model Covariates

| **Variable** | GVIF | DF | GVIF^(1/(2*Df)) |
| --- | --- | --- | --- |
| **SOM clusters** | 1.84 | 4 | 1.08 |
| **Intracranial Volume** | 1.36 | 1 | 1.17 |
| **Sex** | 1.27 | 1 | 1.13 |
| **Child Age** | 1.01 | 1 | 1.13 |
| **Child Race** | 1.77 | 6 | 1.05 |
| **Child Ethnicity** | 1.44 | 1 | 1.20 |
| **Parents’ Highest Level of Education** | 1.48 | 5 | 1.04 |
